# Environmental Toxicant Exposure and Height Among Children and Adolescents

**DOI:** 10.3390/toxics14060481

**Published:** 2026-05-30

**Authors:** Junyu Lu, Jianhui Guo, Yuwan Li, Di Shi, Yaqi Wang, Xinyao Lian, Shuyue Li, Xindou Chen, Shaodan Huang, Jing Guo, Qi Su, Xiaoheng Li, Jing Li

**Affiliations:** 1 Institute of Child and Adolescent Health, School of Public Health, Peking University, Beijing 100191, China; 2010306210@bjmu.edu.cn (J.L.);; 2 National Health Commission Key Laboratory of Reproductive Health, Beijing 100191, China; 3 Shenzhen Center for Disease Control and Prevention, Shenzhen 518055, China; 4 Primary and Secondary School Health Care Center of Huairou District, Beijing 101400, China

**Keywords:** exposome, height, NHANES Subsample A, mediation analysis, sex steroid hormones, heavy metals, volatile organic compounds

## Abstract

Environmental toxicants may affect the height of children and adolescents. However, studies on the toxicological effects based on extensive internal exposure omics are still lacking. This study aimed to identify key toxicants associated with height and assess the mediating role of sex steroid hormones. To this end 1660 participants aged 6–19 years from subsample A in the National Health and Nutrition Examination Survey (NHANES) were included. Exposome was characterized by 58 toxicants within 12 families. After assessment by the exposome-wide association analysis and mixture models, we identified 17 toxicants inversely associated with height-for-age Z-scores (HAZ), predominantly metals and volatile organic compound (VOC) metabolites. Tin exhibited the strongest inverse association (β = −0.261), followed by lead (β = −0.230). The primary contributors to reduced height included tin, lead, the VOC metabolite 2-ATCA, ethylene oxide, and nitrate. Notably, males and younger children were the more susceptible subgroups. Furthermore, mediation analysis revealed that sex steroid hormones, particularly total testosterone and estradiol, mediated 8% to 37% of the associations. These findings suggest that endocrine-related pathways may link toxicant exposure to impaired linear growth, highlighting the necessity of reducing exposure during childhood.

## 1. Introduction

The linear growth during childhood and adolescence reflects the overall health status and future development prospects of the individual [1]. This period represents a particularly sensitive developmental window [2], during which the growth trajectory determines adult height and has a long-term impact on health [3,4]. In addition, height during childhood is also believed to be related to cognitive development and motor skills [5,6]. Therefore, identifying modifiable factors that may adversely affect linear growth during childhood and adolescence is of substantial public health importance.

Although the roles of genes and nutrition in growth have been gradually clarified [7,8], environmental factors are also important factors influencing height growth [9]. Advancements in biomonitoring have revealed that children and adolescents are simultaneously exposed to a complex cocktail of chemicals [10]. However, existing studies on height remain largely confined to a narrow set of legacy pollutants, such as lead and cadmium, leaving the potential impact of a broader spectrum of environmental toxicants largely unexplored. Furthermore, conventional analytic approaches have predominantly focused on single pollutants in isolation, failing to capture the joint, opposing, or nonlinear effects of co-exposures [11]. Consequently, the true effects of combined exposure have often been masked in previous studies [12].

The exposome-wide association study (EWAS) framework provides a holistic paradigm to assess the totality of mixed environmental exposures and their biological consequences across the life course [13,14]. Relevant exposome studies have shown that mixed environmental exposures may affect neurodevelopment and metabolic health in children and adolescents [10,15]. However, exposome research on height growth is still limited. The lack of broad internal exposure measurements has also restricted studies on the links between environmental toxicants and linear growth. Thus, a comprehensive assessment of the exposome is therefore crucial for understanding their associations with height in children and adolescents [16,17].

Moreover, various environmental toxicants, such as heavy metals and VOCs, are recognized as endocrine-disrupting agents. These substances can alter hypothalamic–pituitary–gonadal (HPG) axis activity and perturb sex steroid synthesis [18,19]. Sex hormones, including total testosterone (TT), estradiol (E2), and sex hormone-binding globulin (SHBG), play essential roles in growth-plate regulation, bone mineral accrual, and pubertal maturation [20]. Concentrations of these hormones undergo a dramatic surge during mid-to-late puberty, exhibiting pronounced gender variation [21,22]. Endocrine dysregulation acts as a potent disruptor of skeletal architecture. Deficits stifle chondrocyte proliferation and retard growth-plate maturation, while supraphysiological concentrations precipitate premature epiphyseal fusion [23]. Thus, we posit that endocrine perturbation serves as one of the primary conduits for toxicant-induced growth deficits. This biological plausibility motivates the inclusion of TT, E2, and SHBG as potential mediators in this study.

Leveraging an exposome framework, we systematically examine the associations between a wide range of environmental toxicants and height-for-age Z-scores (HAZ) in U.S. youth. Beyond identifying individual adverse agents, we applied mixture modeling to quantify joint effects and dissected the potential mediating influence of sex steroid hormones by mediation analysis. This integrated approach aims to provide new evidence to support risk assessment and to inform policies and interventions that protect height growth during childhood and adolescence.

## 2. Materials and Methods

### 2.1. Study Population

National Health and Nutrition Examination Survey (NHANES) is a continuous program which implements a complex, stratified, multistage probability sampling design. It combines in-home interviews with standardized mobile examination center visits that include physical measurements and biological specimen collection. Furthermore, NHANES randomly assigns approximately one-third of the general study population to specific subsamples for environmental biomonitoring [24]. In this study, we restricted our analysis to NHANES Subsample A from the 2013–2014 and 2015–2016 cycles, a subsample covering participants aged 6 to 19 years with extensive laboratory measurements of environmental biomarkers. Ultimately, 1660 participants with complete height data were included. Detailed information of sample selection is presented in Figure S1 and Text S1. Ethical clearance was granted by the National Center for Health Statistics (NCHS) Research Ethics Review Board, and all participants provided written informed consent. Our study complies with the Strengthening the Reporting of Observational Studies reporting guideline for cross-sectional studies [25].

### 2.2. Outcome Assessment

HAZ was used as the primary outcome to assess linear growth among children and adolescents. Standing height was measured in centimeters by trained technicians during the NHANES physical examination using a standardized stadiometer. And HAZ was calculated according to the Centers for Disease Control and Prevention (CDC) growth reference for children and adolescents aged 2 to 20 years. All anthropometric indices were calculated from measured height, age in months, and sex using CDC reference curves. The distribution of HAZ in our sample was checked against CDC growth chart percentiles to ensure consistency with national reference standards [26].

### 2.3. Exposure Assessment

Following the household interview, blood and urine specimens were collected using standardized NHANES protocols. We initially identified 79 toxicants across 12 families in Subsample A. After excluding biomarkers with excessive missingness or measurements below the lower limit of detection (LLOD), a total of 58 toxicants within 12 categories were retained.

The analyzed toxicants included hemoglobin-bound reactive organic compounds such as acrylamide, glycidamide, and ethylene oxide (EO), as well as urinary biomarkers of perchlorate, nitrate, thiocyanate, metals, arsenic species, polycyclic aromatic hydrocarbons (PAHs), volatile organic compounds (VOCs), and nicotine metabolites. Laboratory analyses were conducted by CDC for Environmental Health using standardized protocols. Detailed descriptions of toxicant categories, measurement methods, and analytical limits are provided in the Text S2 and Table S2 in Supplementary Materials [27]. Blood biomarker concentrations were normalized to hemoglobin levels. Urinary biomarkers were normalized to urinary creatinine concentration (milligrams per gram creatinine) to account for urine dilution variability [28].

### 2.4. Statistical Analysis

We performed statistical analyses of the exposome following a multistep analytic framework, integrating single-exposure, mediation, mixture, and stratified approaches to evaluate the associations between environmental toxicants and height-related outcomes among children and adolescents.

EWAS analysis was first conducted to systematically examine the independent associations between each environmental toxicant and HAZ, with general linear models (GLMs) assuming Gaussian distribution. Two models were constructed: Model 1 was an unadjusted model to evaluate the crude associations; Model 2 was specified a priori as the primary model to account for major demographic, socioeconomic, nutritional, and behavioral factors. Prespecified covariates included age, sex, race/ethnicity, daily energy intake, weekly physical activity frequency, household smokers, and family income-to-poverty ratio, which were selected based on previous epidemiologic literature and biological plausibility [5,29,30,31]. We adjusted *p*-values using the Benjamini–Hochberg (BH) method to control the false discovery rate (FDR) below 0.05 [32].

We applied Weighted Quantile Sum (WQS) regression and Bayesian Kernel Machine Regression (BKMR) to evaluate the joint effects of the toxicant mixture. First, we applied a WQS regression model to construct an exposure index, where weights represent each toxicant’s relative contribution to the overall effect. The direction of weights and selection of exposure were constrained according to prior toxicological knowledge and the observed association in EWAS analysis [33]. For the WQS analysis, we randomly partitioned the dataset into a training subset (40%) and a testing subset (60%). The model was established using 2000 bootstrap replications. To further explore potential nonlinear exposure–response relationships and mixture effects, we applied a BKMR model. We calculated posterior inclusion probabilities (PIPs) to quantify the relative importance of each toxicant and generated univariate exposure-response functions [34]. All analyses were performed using 20,000 iterations of the Markov Chain Monte Carlo method. BKMR model did not impose directional constraints and incorporated all continuous exposure variables simultaneously.

We implemented a pairwise mediation framework to explore potential biological mechanisms mediated by sex steroid hormones (TT, E2, and SHBG) [35]. Natural direct and indirect effects were estimated under the counterfactual framework using the nonparametric bootstrap method (1000 replications) to obtain 95% confidence intervals.

We performed stratified analyses to evaluate potential effect modification by sex (male vs. female) and age group (6–11 years vs. 12–19 years). Separate EWAS and mediation analysis were fitted within each subgroup using identical specifications and covariate adjustments.

Finally, a sensitivity analysis was conducted to evaluate the potential impacts of the metals that were initially excluded due to high limits of detection. We transformed these metals into binary variables (detected vs. non-detected) and assessed their associations with HAZ using the fully adjusted model.

Additional methodological details are provided in the Text S3 in Supplementary Materials. All analyses were conducted in R (version 4.4.2). The childsds package was used to calculate HAZ, the mediation package for causal mediation analysis, the gWQS package for WQS regression, and the bkmr package for BKMR modeling.

## 3. Results

### 3.1. Participant Characteristics

A total of 1660 participants aged 6–19 years were included, comprising 831 males (50.1%) and 829 females (49.9%). Among them, 786 (47.3%) were aged 6–11 years and 874 (52.7%) were aged 12–19 years, while the median (Q1, Q3) age was 12.00 (9.00–15.00) years. Regarding race and ethnicity, 371 participants (22.3%) were Mexican American, 425 (25.6%) were non-Hispanic White, 390 (23.5%) were non-Hispanic Black, and 474 (28.6%) were of other racial or ethnic backgrounds. The median family poverty-to-income ratio was 1.47 (0.77–2.97). Participants had a median daily energy intake of 1829.75 (1450.25–2287.00) kcal, which was significantly higher among males than females (*p* < 0.001). Physical activity frequency also differed by sex (*p* = 0.008), with approximately 39% of participants engaging in exercise on all 7 days per week. The median height and HAZ were 152.40 (133.80–164.20) cm and 0.37 (−0.46–1.10) respectively. Table 1 presents the more detailed demographic and anthropometric characteristics of the study participants. The baseline data without multiple imputations are shown in Table S1 in Supplementary Materials.

### 3.2. Exposure Characteristics

Among initial 79 toxicants in subsample A, 58 with sufficient data were retained in the main analysis. Detection frequencies (DF) for the retained toxicants ranged from 10% to 100%, with 41 toxicants exhibiting a DF > 90%. Of the 58 retained toxicants, 47 (DF > 70%) were analyzed as continuous variables after natural log transformation, while 11 were analyzed as categorical variables according to their detection frequencies (Table S2 in Supplementary Materials). Distributions of exposure concentrations varied substantially across chemical families. For example, the median urinary concentrations of nitrate, thiocyanate, and perchlorate were higher than those of most other analytes, whereas the median levels of mercury, cadmium, and lead were relatively low but exhibited wide interindividual variation. Pairwise correlations among the 58 toxicants ranged from nearly zero (|r| < 0.01) to 0.947, with stronger intercorrelations observed within the same exposure families, such as among cotinine metabolites, while correlations between different exposure families were generally weak to moderate (Figure S2 in Supplementary Materials).

### 3.3. Association Between Environmental Toxicant Exposure and HAZ

**Figure 1:**
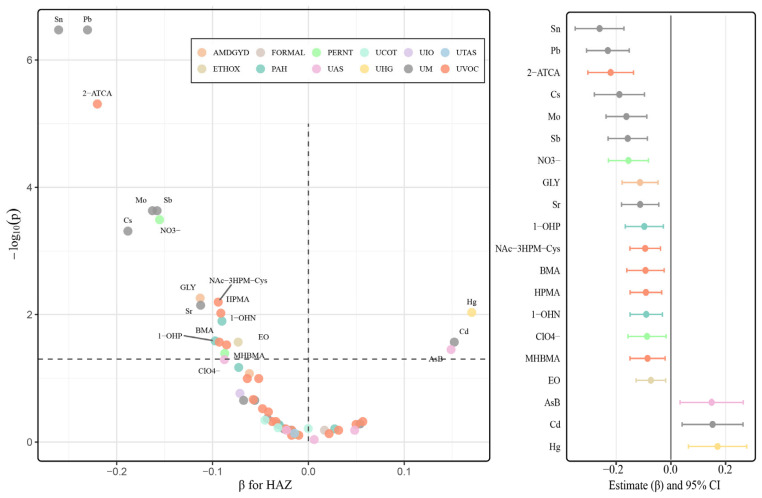
Assessment of relationships between 58 toxicants and HAZ in 1660 children and adolescents (EWAS Analysis). Notes: The left panel displays a volcano plot illustrating the associations between toxicants and HAZ. The horizontal dashed line represents the statistical significance threshold after adjusting for the false discovery rate (FDR < 0.05), and the vertical dashed line indicates an estimate (β) of zero. The right panel shows the estimated β coefficients and 95% CIs for toxicants with significant associations (*p* value < 0.05). All results were derived from Model 2, adjusted for age, sex, race/ethnicity, daily energy intake, weekly physical activity frequency, household smokers, and family income-to-poverty ratio. Abbreviations: AMDGYD, acrylamide and glycidamide; ETHOX, ethylene oxide; FORMAL, formaldehyde; PERNT, perchlorate, nitrate, and thiocyanate; UCOT, cotinine; UIO, iodine; UTAS, total arsenic; UAS, speciated arsenic; UHG, mercury; UM, metal; PAH, polycyclic aromatic hydrocarbon; UVOC, volatile organic compound; Sn, tin; Pb, lead; 2-ATCA, 2-aminothiazoline-4-carboxylic acid; Cs, cesium; Mo, molybdenum; Sb, antimony; NO_3_^−^, nitrate; GLY, glycidamide; Sr, strontium; 1-OHP, 1-hydroxypyrene; NAc-3HPM-Cys, N-acetyl-S-(3-hydroxypropyl-1-methyl)-L-cysteine; BMA, N-acetyl-S-(benzyl)-L-cysteine; HPMA, N-acetyl-S-(2-hydroxypropyl)-L-cysteine; 1-OHN, 1-hydroxynaphthalene; ClO_4_^−^, perchlorate; MHBMA, N-acetyl-S-(4-hydroxy-2-butenyl)-L-cysteine; EO, ethylene oxide; AsB, arsenobetaine; Cd, cadmium; Hg, mercury.

EWAS identified significant inverse associations between HAZ and metals (6 types), VOC metabolites (5 types), PAH metabolites (2 types), perchlorate and nitrate compounds, ethylene oxide, and glycidamide (Figure 1, Table 2). Notably, metals and VOC metabolites represented the families with the greatest number of significant toxicants. In the unadjusted model (Model 1), only a small number of toxicants showed statistically significant associations with HAZ after FDR correction. Specifically, CEMA, DHBMA, PGA, iodine, and strontium were associated with HAZ, with strontium showing an inverse association and the others showing positive associations. In the fully adjusted model (Model 2), the strongest inverse association was observed for tin, with each IQR increase in log-transformed concentration corresponding to a 0.26-unit decrease in HAZ (β = −0.261; *p* < 0.001), followed by lead (Pb; β = −0.230; *p* < 0.001). Comparable strong inverse associations were observed for 2-aminothiazoline-4-carboxylic acid (2-ATCA; β = −0.220; *p* < 0.001) and nitrate (β = −0.155; *p* < 0.001). In contrast, positive associations were observed for three toxicants: mercury (β = 0.170; *p* = 0.0093), cadmium (β = 0.152; *p* = 0.027), and arsenobetaine (β = 0.149; *p* = 0.035). Primary associations remained robust in our sensitivity analysis (Figure S3, Table S3). We retained toxicants with extremely low detection rates and modeled them as binary variables. None of these substances showed significant associations with HAZ.

### 3.4. Joint Associations Between Mixed Environmental Toxicant Exposure and HAZ

**Figure 2:**
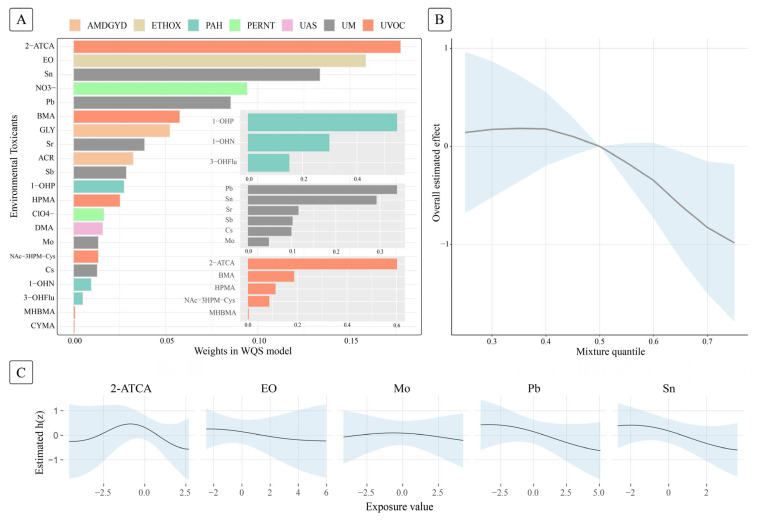
Integrated WQS regression and BKMR model mixture analyses. Notes: Panel (**A**) shows the estimated weights of individual toxicants and specific chemical families from the WQS regression model. Panel (**B**) illustrates the overall effect of the toxicant mixture on HAZ estimated by the BKMR model, and the blue shaded areas represent the pointwise 95% credible intervals. Panel (**C**) presents the univariate exposure–response functions for the top contributing toxicants from the BKMR model, and the blue shaded areas represent the pointwise 95% credible intervals. All results were adjusted for age, sex, race/ethnicity, daily energy intake, weekly physical activity frequency, household smokers, and family income-to-poverty ratio. Abbreviations: AMDGYD, acrylamide and glycidamide; ETHOX, ethylene oxide; PAH, polycyclic aromatic hydrocarbon; PERNT, perchlorate, nitrate, and thiocyanate; UAS, speciated arsenic; UM, metal; UVOC, volatile organic compound; 1-OHN, 1-hydroxynaphthalene; 1-OHP, 1-hydroxypyrene; 2-ATCA, 2-aminothiazoline-4-carboxylic acid; 3-OHFlu, 3-hydroxyfluorene; ACR, acrylamide; BMA, N-acetyl-S-(benzyl)-L-cysteine; ClO_4_^−^, perchlorate; Cs, cesium; CYMA, N-acetyl-S-(2-cyanoethyl)-L-cysteine; DMA, dimethylarsinic acid; EO, ethylene oxide; GLY, glycidamide; HPMA, N-acetyl-S-(2-hydroxypropyl)-L-cysteine; MHBMA, N-acetyl-S-(4-hydroxy-2-butenyl)-L-cysteine; Mo, molybdenum; NAc-3HPM-Cys, N-acetyl-S-(3-hydroxypropyl-1-methyl)-L-cysteine; NO_3_^−^, nitrate; Pb, lead; Sb, antimony; Sn, tin; Sr, strontium.

The WQS regression model was applied to evaluate the cumulative burden of the toxicant mixture. After adjusting for all prespecified covariates, the overall WQS index showed a significant inverse association with HAZ (Figure S4). Among individual toxicants, 2-aminothiazoline-4-carboxylic acid (2-ATCA; weight = 0.178), ethylene oxide (EO; 0.159), tin (Sn; 0.134), and nitrate (NO_3_^−^; 0.094) were the primary contributors to the index. Family-specific models demonstrated consistent weight patterns (Figure 2A, Table S3). The BKMR model corroborated these findings: the overall exposure–response function indicated a monotonic decline in HAZ as the mixture quantiles increased. Shifting all exposure components from median levels to 75th percentile was associated with an estimated 0.61-unit reduction in HAZ (Figure 2B). Furthermore, the BKMR PIP rankings aligned closely with the WQS weights, with 2-ATCA, Sn, NO_3_^−^, Mo, EO, and Pb identified as the most influential contributors (Tables S4 and S5). Notably, these high-PIP toxicants all exhibited similarly negative and nonlinear exposure–response patterns in the univariate BKMR functions (Figure 2C). Univariate exposure–response plots for the remaining toxicants are presented in Figure S5.

### 3.5. Heterogeneity of Associations Across Subgroups Stratified by Age and Sex

**Figure 3:**
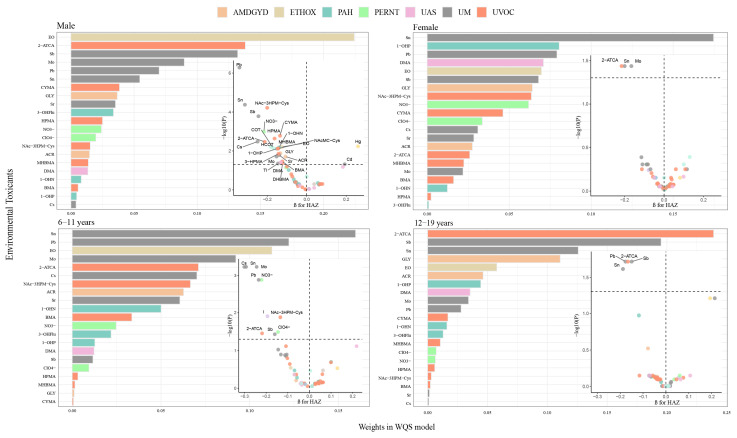
Assessment of single and mixed relationships between toxicants and HAZ in 1660 children and adolescents stratified by age and sex. Notes: The figure displays the estimated toxicant weights from WQS regression (left bar charts) and individual association results from EWAS (right inset volcano plots) across subgroups. In the volcano plots, the horizontal dashed line indicates FDR significance threshold of 0.05, and the vertical dashed line indicates an estimate (β) of zero. All models were adjusted for race/ethnicity, daily energy intake, weekly physical activity frequency, household smokers, family income-to-poverty ratio, and either age (for sex-stratified models) or sex (for age-stratified models). Abbreviations: AMDGYD, acrylamide and glycidamide; ETHOX, ethylene oxide; PAH, polycyclic aromatic hydrocarbon; PERNT, perchlorate, nitrate, and thiocyanate; UAS, speciated arsenic; UM, metal; UVOC, volatile organic compound; 1-OHN, 1-hydroxynaphthalene; 1-OHP, 1-hydroxypyrene; 2-ATCA, 2-aminothiazoline-4-carboxylic acid; 3-OHFlu, 3-hydroxyfluorene; ACR, acrylamide; BMA, N-acetyl-S-(benzyl)-L-cysteine; Cd, cadmium; ClO_4_^−^, perchlorate; COT, total cotinine; Cs, cesium; CYMA, N-acetyl-S-(2-cyanoethyl)-L-cysteine; DHBMA, N-acetyl-S-(3,4-dihydroxybutyl)-L-cysteine; DMA, dimethylarsinic acid; EO, ethylene oxide; GLY, glycidamide; HCOT, total hydroxycotinine; Hg, mercury; HPMA, N-acetyl-S-(2-hydroxypropyl)-L-cysteine; I, iodine; MHBMA, N-acetyl-S-(4-hydroxy-2-butenyl)-L-cysteine; Mo, molybdenum; NAc-3HPM-Cys, N-acetyl-S-(3-hydroxypropyl-1-methyl)-L-cysteine; NAcMC-Cys, N-acetyl-S-(N-methylcarbamoyl)-L-cysteine; NO_3_^−^, nitrate; Pb, lead; Sb, antimony; Sn, tin; Sr, strontium; Tl, thallium.

Stratified analyses revealed substantial heterogeneity across subgroups (Figure 3, Table S6). In the EWAS models, males and younger children (25 and 10 types) exhibited a larger number of significant associations with HAZ compared with females and adolescents (3 and 4 types). WQS weights highlighted distinct primary drivers within these groups. In males, EO received the highest weight, followed by 2-ATCA and Sb. Notably, the top-ranked EO in males dropped to fifth place in females. In contrast, Sn was the dominant contributor in females, followed by 1-OHP, both of which fell outside the top five in males. Age-stratified models also demonstrated shifting exposure priorities. Sn and Pb were the top weighted exposures in children aged 6 to 11 years. However, 2-ATCA became the leading contributor in the adolescents group, with GLY emerging as an additional contributor. Despite these differences, Pb, Sn, and 2-ATCA demonstrated consistent relevance across most strata.

### 3.6. Analysis of the Mediating Role of TT, E2 and SHBG

**Figure 4:**
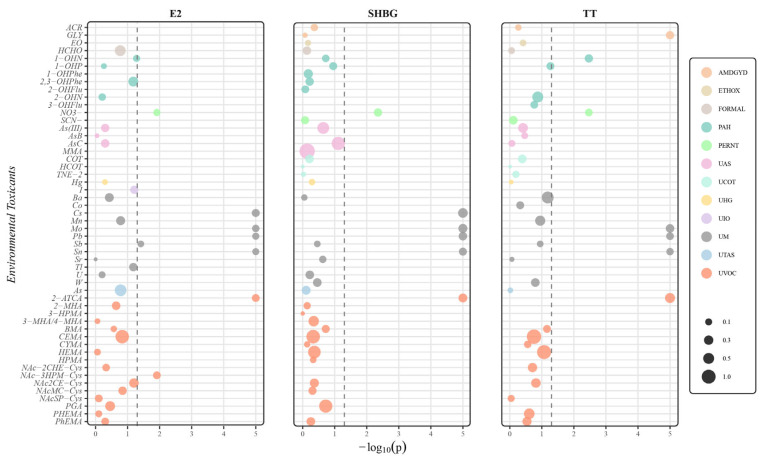
Pairwise mediation of the association between toxicants and HAZ by sex hormones. Notes: The plots illustrate the significance of the natural indirect effects of individual environmental toxicants on HAZ, mediated by three sex steroid hormones. The vertical dashed line indicates a mediation significance threshold of *p* = 0.05. The size of each circle is proportional to the proportion of the total effect mediated by the respective hormone. All mediation models were adjusted for age, sex, race/ethnicity, daily energy intake, weekly physical activity frequency, number of household smokers, and family income-to-poverty ratio. Abbreviations: AMDGYD, acrylamide and glycidamide; ETHOX, ethylene oxide; FORMAL, formaldehyde; PAH, polycyclic aromatic hydrocarbon; PERNT, perchlorate, nitrate, and thiocyanate; UAS, speciated arsenic; UCOT, cotinine; UHG, mercury; UIO, iodine; UM, metal; UTAS, total arsenic; UVOC, volatile organic compound; E2, estradiol; SHBG, sex hormone-binding globulin; TT, total testosterone; 1-OHN, 1-hydroxynaphthalene; 1-OHP, 1-hydroxypyrene; 1-OHPhe, 1-hydroxyphenanthrene; 2,3-OHPhe, 2- and 3-hydroxyphenanthrene; 2-ATCA, 2-aminothiazoline-4-carboxylic acid; 2-MHA, 2-methylhippuric acid; 2-OHFlu, 2-hydroxyfluorene; 2-OHN, 2-hydroxynaphthalene; 3-HPMA, N-acetyl-S-(3-hydroxypropyl)-L-cysteine; 3-MHA/4-MHA, 3-methylhippuric acid and 4-methylhippuric acid; 3-OHFlu, 3-hydroxyfluorene; ACR, acrylamide; As, total arsenic; As(III), arsenous acid; AsB, arsenobetaine; AsC, arsenocholine; Ba, barium; BMA, N-acetyl-S-(benzyl)-L-cysteine; CEMA, N-acetyl-S-(2-carboxyethyl)-L-cysteine; Co, cobalt; COT, total cotinine; Cs, cesium; CYMA, N-acetyl-S-(2-cyanoethyl)-L-cysteine; EO, ethylene oxide; GLY, glycidamide; HCHO, formaldehyde; HCOT, total hydroxycotinine; HEMA, N-acetyl-S-(2-hydroxyethyl)-L-cysteine; Hg, mercury; HPMA, N-acetyl-S-(2-hydroxypropyl)-L-cysteine; I, iodine; MMA, monomethylarsonic acid; Mn, manganese; Mo, molybdenum; NAc-2CHE-Cys, N-acetyl-S-(2-carbamoyl-2-hydroxyethyl)-L-cysteine; NAc-3HPM-Cys, N-acetyl-S-(3-hydroxypropyl-1-methyl)-L-cysteine; NAc2CE-Cys, N-acetyl-S-(2-carbamoylethyl)-L-cysteine; NAcMC-Cys, N-acetyl-S-(N-methylcarbamoyl)-L-cysteine; NAcSP-Cys, N-acetyl-S-(n-propyl)-L-cysteine; NO_3_^−^, nitrate; Pb, lead; PGA, phenylglyoxylic acid; PHEMA, N-acetyl-S-(phenyl)-L-cysteine; PhEMA, N-acetyl-S-(phenyl-2-hydroxyethyl)-L-cysteine; Sb, antimony; SCN^−^, thiocyanate; Sn, tin; Sr, strontium; Tl, thallium; TNE-2, total nicotine equivalent-2; U, uranium; W, tungsten.

To further investigate potential endocrine pathways underlying the associations between environmental toxicants and linear growth, mediation analyses were conducted with TT, E2, and SHBG as mediators (Figure 4, Table S7). A total of 81 mediation models showed statistically significant indirect effects, of which 21 (25.9%) were partial mediation models with both significant indirect (ACME) and direct (ADE) effects. Across the three hormonal mediators, the patterns of mediation were largely consistent. Overall, nitrate, Pb, Sn, Mo, Cs, and 2-ATCA consistently exhibited significant indirect pathways across all three hormonal mediators. The proportions of mediation in these partial models ranged approximately from 8% to 37%. Notably, the pathway of 2-ATCA mediated by TT showed the largest proportion mediated (approximately 37%). In this pathway, the ACME estimate of –0.082 indicates that a one-IQR increase in log-transformed 2-ATCA concentrations is expected to reduce HAZ by 0.082 units through its effect on TT.

## 4. Discussion

In this nationally representative study, we identified 17 toxicants across 6 families that were inversely associated with HAZ, among which males and children younger than 12 years exhibited greater susceptibility to environmental toxicant exposure. Sex steroid hormones (TT, E2, and SHBG) were estimated to mediate 8% to 37% of the pathways linking environmental toxicants with reduced linear growth. These findings underscore the importance of protective measures targeting susceptible subgroups to preserve normal physically developmental trajectories.

Among the identified toxicants, 2-ATCA, lead (Pb), tin (Sn), nitrate, and ethylene oxide (EO) emerged as the primary contributors to reduce linear growth. Metals, in particular, exhibited the most consistent adverse associations across both single-exposure and mixture models, aligning with prior epidemiological evidence regarding heavy metals and growth deficits [36]. Lead has historically been linked to growth impairment, mechanistically attributed to the suppression of the insulin-like growth factor 1 (GH-IGF-1) axis and disruption of chondrocyte proliferation [37,38]. Similarly, tin may interfere with bone remodeling through endocrine disruption and oxidative stress pathways, supporting the biological plausibility of the observed metal–growth associations [37,39]. The prominent contribution of metals such as tin may partly reflect common exposure sources in children and adolescents, including dietary intake from canned foods, migration from materials that come into contact with food, and the use of tin compounds in PVC materials [40]. Organotin compounds have also been detected in household dust, suggesting that indoor dust ingestion may represent another plausible exposure route [41].

Recent epidemiological studies have linked VOC exposure to altered growth indicators in adolescents [42]. Representative VOC metabolites, such as 2-ATCA and BMA, have been shown in previous studies to affect the height of children and adolescents through disruption of the GH-IGF-1 axis and thyroid hormone signaling [43], both of which are critical for chondrocyte proliferation and epiphyseal plate activity. The relatively high internal exposure to VOCs in daily life through both environmental and dietary routes may partially explain its strong contribution in the mixture analyses [44]. Likewise, PAHs are known to activate the aryl hydrocarbon receptor and provoke oxidative stress and inflammation, which can impair osteoblast function and alter endocrine axes relevant to growth [45]. Therefore, our findings partly align with studies demonstrating that NIS inhibitors can impair bone maturation and linear growth. Previous mechanistic and epidemiologic evidence indicates that inorganic anions like nitrate and perchlorate competitively inhibit the sodium–iodide symporter (NIS), thereby reducing thyroid hormone synthesis [46]. Thyroid hormones are essential regulators of chondrocyte differentiation, bone maturation, and systemic metabolic processes that support linear growth; disruption of thyroid hormone production during childhood can therefore impair skeletal development and height accrual [47].

The stratified analyses revealed meaningful effect heterogeneity. Children aged 6–11 years exhibited a larger number and magnitude of significant associations than adolescents. Substances such as nitrate, Pb, and EO present stronger effects on younger children due to their immature detoxification capacities. Consequently, oxidatively active agents display a distinct tendency of compromising the integrity of the thyroid and IGF-1 pathways [48,49]. Furthermore, these age-related differences likely reflect a shift in exposure sources. The dominance of Sn and Pb likely stems from hand-to-mouth behaviors, which can increase ingestion of dust and soil contaminated by heavy metals [50,51]. Conversely, the prominence of 2-ATCA, GLY, and antimony in adolescents suggests a shift from surface-related environmental exposure to more lifestyle-related sources, specifically for active or passive smoking, consumption of fried foods, and the use of plastic food packaging [52,53,54]. The negative association between iodine exposure and HAZ observed only in children aged 6–11 years aligns with prior evidence. Excess iodine intake in early childhood can transiently suppress thyroid peroxidase activity, leading to subclinical hypothyroidism [55]. This effect is less pronounced in adolescents, whose thyroid autoregulation is more mature and iodine stores are larger [56].

Similarly, males showed stronger associations than females. The predominance of EO in males warrants specific attention. EO is a potent alkylating agent that induces DNA damage and oxidative stress [57]. Estrogens exhibit antioxidant properties and upregulate the expression of antioxidant enzymes [58]. Therefore, estrogen may partly mitigate oxidative stress-induced damage. This also explains why fewer toxicants showed significant associations among females. Additionally, sex-specific differences in the expression of hepatic drug-metabolizing enzymes contribute to heightened male susceptibility [59]. Previous studies have shown that acrylamide can impair Leydig-cell steroidogenesis and suppress testosterone synthesis. In addition, pediatric mixture analyses suggest that lead-dominant metal exposure patterns are also related to altered SHBG and estradiol among adolescent boys [60,61], lending further support to the endocrine-mediated interpretation of our findings.

This sex-specific susceptibility is partially supported by our mediation analyses. Several key toxicants, including nitrate, Sn, Mo, ACR, GLY, Pb, and especially 2-ATCA, exhibited significant indirect effects through one or more sex hormone mediators. Prior studies have documented that Pb, nitrate, acrylamide-related metabolites, and EO-derived electrophiles can disrupt steroidogenesis, reduce testosterone synthesis, and impair pubertal progression in males [62,63], offering a coherent mechanistic explanation for the observed male-specific burden.

We failed to replicate the inhibitory links reported for arsenobetaine, mercury, and cadmium. Although exhibiting weak positive associations in EWAS analysis, existing evidence does not support a growth-promoting role for these species [64,65,66]. These counterintuitive findings likely reflect residual confounding or chance rather than true biological benefits. For mercury and arsenobetaine, the weak positive associations may partly reflect dietary confounding, as both biomarkers are often related to fish or seafood intake. Higher seafood consumption may also reflect better diet quality, higher intake of high-quality protein and long-chain n-3 fatty acids, rather than beneficial effects of mercury or arsenobetaine [67,68]. Additionally, our study observed no significant associations for several established growth inhibitors, particularly perchlorate and cotinine. This may indicate that urinary burdens did not reach the biological threshold required to produce measurable growth impairment. Furthermore, the robust effects of dominant toxicant could eclipse subtle impacts of low-level cadmium or arsenic exposure within this specific cohort.

### Strengths and Limitations

To our knowledge, our research is one of the largest biomarker-exposome studies focusing on height. It examined the relationships between a relatively comprehensive set of biomarkers of toxicant exposure (12 categories and 58 analytes) and height. The EWAS framework enabled us to systematically screen various chemicals. It helped identify potential risk factors that might be overlooked in traditional single-pollutant studies. We also quantified mixture contributions and explored hormone-mediated pathways, providing a more comprehensive understanding of how environmental exposures may influence linear growth during critical developmental periods.

Several limitations should be considered. First, cross-sectional studies yield a lower level of causal evidence, and temporal relationships between exposures, hormonal changes, and linear growth cannot be conclusively established. The mediation analysis is therefore exploratory, and prospective cohort studies with repeated exposure and hormonal measurements are necessary to validate the proposed pathways. Second, while BKMR allows for interaction assessment, systematic evaluation of pairwise interactions was not feasible given the high dimensionality of the 58-toxicant mixture. We prioritized the estimation of cumulative mixture effects and independent toxicant contributions. Third, the use of NHANES Subsample A imposed limitations on available variables and toxicant coverage. Detailed information regarding individual genetic backgrounds and other critical covariates, specific nutrient intake, duration of sun exposure, and geographic location was unavailable [69,70,71]. Consequently, it is difficult to further evaluate the detailed roles of genetic, nutritional, and environmental factors, as well as urban or rural differences and the geographic distribution of toxicant profiles. In addition, several toxicants potentially relevant to growth, such as polyfluoroalkyl substances, polychlorinated biphenyls, organochlorine pesticides, and carbamate pesticides, were not included in this subsample. More detailed studies are needed to better characterize potential interplay with environmental toxicants in relation to linear growth. Fourth, the single-point biological sample may not accurately capture chronic exposure patterns, particularly for compounds with rapid elimination rates. This may introduce exposure misclassification and bias associations toward the null. Finally, several toxicants had a high proportion of values below the limit of detection. Although we evaluated these high-LOD toxicants through a binary sensitivity analysis, categorizing continuous exposures inevitably loses dose–response information. Therefore, these specific effect estimates might not fully capture the true biological associations.

## 5. Conclusions

In this exposome-based study of US children and adolescents, 17 environmental toxicants in 6 categories were inversely associated with linear growth. Notably, 2-ATCA, EO and Sn emerged as the primary contributors to these adverse effects. Susceptibility to these exposures appeared more pronounced among younger children and males. Detrimental effects of crucial toxicants may operate, in part, through the dysregulation of sex steroid hormones, particularly TT and E2. These findings highlight the potential growth-suppressing role of mixed environmental toxicants and underscore the importance of targeted strategies to reduce exposures during critical developmental windows.

## Figures and Tables

**Table 1:** Participant Characteristics.

Characteristic	Overall	Male	Female
Age group ^a^			
6–11 years	786 (47.3)	398 (47.9)	388 (46.8)
12–19 years	874 (52.7)	433 (52.1)	441 (53.2)
Age ^b^	12.00 (9.00, 15.00)	12.00 (9.00, 15.00)	12.00 (9.00, 16.00)
Sex ^a^			
Male	831 (50.1)	831 (100.0)	-
Female	829 (49.9)	-	829 (100.0)
Race ^a^			
Mexican American	371 (22.3)	166 (20.0)	205 (24.7)
Other Hispanic	197 (11.9)	96 (11.6)	101 (12.2)
Non-Hispanic White	425 (25.6)	230 (27.7)	195 (23.5)
Non-Hispanic Black	390 (23.5)	201 (24.2)	189 (22.8)
Non-Hispanic Asian	160 (9.6)	85 (10.2)	75 (9.0)
Other Race—Including Multi-Racial	117 (7.0)	53 (6.4)	64 (7.7)
Poverty income ratio ^b^	1.47 (0.77, 2.97)	1.52 (0.84, 3.18)	1.46 (0.75, 2.82)
Energy (kcal) ^b^	1829.75 (1450.25, 2287.00)	1955.00 (1596.00, 2474.75)	1694.50 (1330.50, 2093.50)
Physical activity ^a^			
0	47 (2.8)	15 (1.8)	32 (3.9)
1	99 (6.0)	56 (6.7)	43 (5.2)
2	137 (8.3)	61 (7.3)	76 (9.2)
3	175 (10.5)	84 (10.1)	91 (11.0)
4	132 (8.0)	62 (7.5)	70 (8.4)
5	330 (19.9)	150 (18.1)	180 (21.7)
6	93 (5.6)	49 (5.9)	44 (5.3)
7	647 (39.0)	354 (42.6)	293 (35.3)
Household smokers ^a^			
0	1249 (75.2)	617 (74.2)	632 (76.2)
1	269 (16.2)	143 (17.2)	126 (15.2)
2	116 (7.0)	57 (6.9)	59 (7.1)
3	26 (1.6)	14 (1.7)	12 (1.4)
Height (cm) ^b^	152.40 (133.80, 164.20)	152.60 (133.00, 170.25)	152.30 (134.90, 160.70)
HAZ ^b^	0.37 (−0.46, 1.10)	0.47 (−0.34, 1.19)	0.29 (−0.51, 1.04)
TT ^c^	1.04 (0.66)	1.24 (0.81)	0.85 (0.37)
E2 ^c^	0.86 (0.55)	0.67 (0.42)	1.05 (0.60)
SHBG ^c^	3.94 (0.68)	3.92 (0.71)	3.96 (0.65)

^a^ Frequency and percentage [n (%)] are used to describe categorical variables. ^b^ Median and interquartile range [median (Q1, Q3)] are used to describe variables with skewed distributions. ^c^ Mean and standard deviation [mean (SD)] are used to describe continuous variables with a normal distribution. HAZ, Height-for-age Z-score; TT, total testosterone; E2, estradiol; SHBG, sex hormone-binding globulin.

**Table 2:** Associations Between 58 Environmental Toxicants and Height-for-Age Z-Score in US Children and Adolescents (NHANES 2013–2016).

Exposure Family by Toxicant Exposure	Median (Q1,Q3)	Process	Model 1 ^a^		Model 2 ^b^	
			β (95% CI)	*p* Value ^c^	β (95% CI)	*p* Value ^c^
UVOC						
2-MHA	0.212 (0.121–0.436)	log e	−0.032 (−0.105, 0.041)	0.56	−0.038 (−0.106, 0.031)	0.48
3-MHA/4-MHA	1.414 (0.875–2.789)	log e	0.004 (−0.067, 0.074)	0.97	−0.024 (−0.090, 0.042)	0.62
NAc2CE-Cys	0.511 (0.350–0.763)	log e	0.012 (−0.055, 0.080)	0.84	−0.034 (−0.097, 0.030)	0.48
NAcMC-Cys	0.684 (0.482–0.939)	log e	−0.026 (−0.089, 0.038)	0.57	−0.042 (−0.103, 0.019)	0.34
2-ATCA	2.265 (1.105–4.013)	log e	0.086 (0.014, 0.157)	0.13	−0.220 (−0.304, −0.137)	<0.001
BMA	0.065 (0.041–0.108)	log e	0.056 (−0.012, 0.125)	0.34	−0.093 (−0.161, −0.025)	0.027
NAcSP-Cys	NA	2 categories	−0.066 (−0.187, 0.055)	0.53	−0.018 (−0.132, 0.096)	0.78
CEMA	0.828 (0.548–1.267)	log e	0.124 (0.054, 0.193)	0.0097	−0.010 (−0.077, 0.057)	0.78
CYMA	0.014 (0.009–0.027)	log e	−0.040 (−0.091, 0.011)	0.34	−0.052 (−0.102, −0.002)	0.1
DHBMA	3.237 (2.419–4.229)	log e	0.106 (0.040, 0.173)	0.02	−0.057 (−0.126, 0.011)	0.22
NAc-2CHE-Cys	NA	2 categories	0.052 (−0.061, 0.164)	0.56	0.057 (−0.049, 0.163)	0.48
HEMA	NA	2 categories	0.090 (−0.022, 0.202)	0.34	0.022 (−0.084, 0.127)	0.74
HPMA	0.260 (0.172–0.417)	log e	−0.014 (−0.075, 0.047)	0.77	−0.091 (−0.149, −0.034)	0.0095
3-HPMA	2.197 (1.423–3.312)	log e	0.032 (−0.036, 0.101)	0.56	−0.048 (−0.114, 0.018)	0.3
MA	1.163 (0.847–1.628)	log e	0.037 (−0.027, 0.100)	0.51	−0.064 (−0.126, −0.002)	0.1
MHBMA	0.044 (0.029–0.072)	log e	0.031 (−0.036, 0.098)	0.56	−0.085 (−0.150, −0.021)	0.03
PhEMA	NA	2 categories	0.092 (−0.021, 0.204)	0.34	0.050 (−0.056, 0.155)	0.53
PGA	1.920 (1.433–2.609)	log e	0.114 (0.052, 0.175)	0.0081	−0.017 (−0.079, 0.045)	0.65
PHEMA	NA	2 categories	0.068 (−0.043, 0.180)	0.46	0.032 (−0.073, 0.136)	0.65
NAc-3HPM-Cys	1.867 (1.387–2.590)	log e	0.016 (−0.041, 0.072)	0.71	−0.094 (−0.150, −0.038)	0.0064
UTAS						
As	0.054 (0.036–0.098)	log e	0.029 (−0.036, 0.094)	0.56	−0.014 (−0.080, 0.051)	0.73
UM						
Ba	0.013 (0.007–0.024)	log e	0.036 (−0.035, 0.108)	0.56	−0.025 (−0.095, 0.045)	0.62
Cd	NA	2 categories	−0.044 (−0.157, 0.070)	0.59	0.152 (0.041, 0.264)	0.027
Co	0.006 (0.004–0.008)	log e	0.056 (−0.014, 0.126)	0.34	−0.043 (−0.114, 0.028)	0.43
Cs	0.045 (0.032–0.063)	log e	0.084 (0.008, 0.161)	0.16	−0.188 (−0.280, −0.097)	<0.001
Mo	0.587 (0.392–0.866)	log e	0.053 (−0.017, 0.123)	0.35	−0.163 (−0.237, −0.088)	<0.001
Mn	NA	2 categories	0.081 (−0.035, 0.197)	0.4	0.054 (−0.055, 0.163)	0.52
Pb	0.002 (0.002–0.004)	log e	0.025 (−0.046, 0.097)	0.63	−0.230 (−0.308, −0.152)	<0.001
Sb	0.001 (0.000–0.001)	log e	0.036 (−0.034, 0.105)	0.56	−0.158 (−0.230, −0.086)	<0.001
Sn	0.007 (0.003–0.017)	log e	0.063 (−0.018, 0.144)	0.34	−0.261 (−0.350, −0.172)	<0.001
Sr	0.912 (0.530–1.478)	log e	−0.113 (−0.183, −0.042)	0.02	−0.112 (−0.180, −0.044)	0.0071
Tl	0.002 (0.001–0.003)	log e	0.086 (0.011, 0.162)	0.15	−0.068 (−0.150, 0.015)	0.22
W	0.001 (0.001–0.002)	log e	0.084 (0.015, 0.153)	0.13	−0.056 (−0.125, 0.013)	0.22
U	0.000 (0.000–0.000)	log e	−0.022 (−0.090, 0.046)	0.66	−0.032 (−0.097, 0.034)	0.53
UIO						
I	1.499 (0.909–2.633)	log e	0.142 (0.068, 0.215)	0.0081	−0.071 (−0.151, 0.008)	0.17
UHG						
Hg	NA	2 categories	0.078 (−0.035, 0.190)	0.4	0.170 (0.064, 0.277)	0.0093
UCOT						
COT	0.005 (0.002–0.028)	log e	−0.009 (−0.078, 0.060)	0.9	−0.032 (−0.109, 0.046)	0.6
HCOT	0.008 (0.003–0.052)	log e	0.003 (−0.067, 0.073)	0.97	−0.045 (−0.124, 0.033)	0.46
TNE-2	NA	log e	−0.000 (−0.001, −0.000)	0.16	−0.000 (−0.001, 0.000)	0.61
UAS						
As(III)	0.004 (0.002–0.007)	log e	−0.005 (−0.080, 0.071)	0.97	−0.023 (−0.095, 0.049)	0.65
AsB	NA	2 categories	0.106 (−0.016, 0.228)	0.34	0.149 (0.033, 0.264)	0.035
AsC	NA	2 categories	−0.005 (−0.168, 0.158)	0.97	0.048 (−0.104, 0.200)	0.65
DMA	0.035 (0.023–0.055)	log e	−0.002 (−0.071, 0.067)	0.97	−0.088 (−0.160, −0.015)	0.051
MMA	NA	2 categories	−0.082 (−0.198, 0.034)	0.4	0.006 (−0.104, 0.116)	0.91
PERNT						
ClO4-	0.031 (0.019–0.053)	log e	0.079 (0.013, 0.146)	0.13	−0.087 (−0.157, −0.018)	0.041
NO3-	522.078 (383.974–718.824)	log e	0.029 (−0.039, 0.096)	0.56	−0.155 (−0.228, −0.082)	<0.001
SCN-	10.315 (5.789–17.553)	log e	0.061 (−0.010, 0.132)	0.34	−0.012 (−0.080, 0.055)	0.76
PAH						
1-OHN	8.862 (5.353–16.714)	log e	−0.027 (−0.089, 0.036)	0.56	−0.090 (−0.149, −0.031)	0.013
2-OHN	51.600 (28.800–90.488)	log e	0.006 (−0.070, 0.082)	0.97	0.027 (−0.046, 0.101)	0.62
3-OHFlu	0.719 (0.473–1.163)	log e	0.019 (−0.048, 0.086)	0.71	−0.073 (−0.136, −0.009)	0.068
2-OHFlu	1.528 (1.087–2.333)	log e	0.043 (−0.021, 0.108)	0.4	−0.018 (−0.080, 0.044)	0.65
1-OHPhe	0.909 (0.610–1.350)	log e	0.065 (−0.001, 0.132)	0.25	−0.019 (−0.084, 0.046)	0.65
1-OHP	1.510 (0.971–2.428)	log e	0.030 (−0.040, 0.100)	0.56	−0.097 (−0.167, −0.028)	0.026
2,3-OHPhe	1.152 (0.812–1.721)	log e	0.086 (0.018, 0.155)	0.13	−0.030 (−0.097, 0.036)	0.54
FORMAL						
HCHO	131.000 (121.000–143.000)	log e	0.001 (−0.059, 0.062)	0.97	0.017 (−0.039, 0.073)	0.65
ETHOX						
EO	20.400 (16.270–27.910)	log e	−0.036 (−0.092, 0.021)	0.44	−0.073 (−0.127, −0.019)	0.027
AMDGYD						
ACR	41.400 (33.900–50.900)	log e	−0.048 (−0.109, 0.012)	0.34	−0.062 (−0.118, −0.005)	0.084
GLY	39.900 (31.000–52.000)	log e	−0.033 (−0.102, 0.036)	0.56	−0.113 (−0.178, −0.047)	0.0055

Note: ^a^ Unadjusted model. ^b^ Constructed by adjusting for age, sex, race/ethnicity, daily energy intake, weekly physical activity frequency, numbers of family smokers and family income-to-poverty ratio. ^c^ Adjusted to control the false discovery rate at 5%. Abbreviations: AMDGYD, acrylamide and glycidamide; ETHOX, ethylene oxide; FORMAL, formaldehyde; UIO, iodine; PERNT, perchlorate, nitrate, and thiocyanate; UM, metals; PAH, polycyclic aromatic hydrocarbon; UHG, mercury; UVOC, volatile organic compound; UTAS, total arsenic; UAS, speciated arsenic; UCOT, cotinine. ACR, acrylamide; GLY, glycidamide; EO, ethylene oxide; HCHO, formaldehyde; I, iodine; ClO_4_^−^, perchlorate; NO_3_^−^, nitrate; SCN^−^, thiocyanate; Ba, barium; Cd, cadmium; Co, cobalt; Cs, cesium; Mo, molybdenum; Mn, manganese; Pb, lead; Sb, antimony; Sn, tin; Sr, strontium; Tl, thallium; W, tungsten; U, uranium; 1-OHN, 1-hydroxynaphthalene; 2-OHN, 2-hydroxynaphthalene; 3-OHFlu, 3-hydroxyfluorene; 2-OHFlu, 2-hydroxyfluorene; 1-OHPhe, 1-hydroxyphenanthrene; 2,3-OHPhe, 2- and 3-hydroxyphenanthrene; 1-OHP, 1-hydroxypyrene; Hg, mercury; 2-MHA, 2-methylhippuric acid; 3-MHA/4-MHA, 3-methylhippuric acid and 4-methylhippuric acid; NAc2CE-Cys, N-acetyl-S-(2-carbamoylethyl)-L-cysteine; NAcMC-Cys, N-acetyl-S-(N-methylcarbamoyl)-L-cysteine; 2-ATCA, 2-aminothiazoline-4-carboxylic acid; BMA, N-acetyl-S-(benzyl)-L-cysteine; NAcSP-Cys, N-acetyl-S-(n-propyl)-L-cysteine; CEMA, N-acetyl-S-(2-carboxyethyl)-L-cysteine; CYMA, N-acetyl-S-(2-cyanoethyl)-L-cysteine; DHBMA, N-acetyl-S-(3,4-dihydroxybutyl)-L-cysteine; NAc-2CHE-Cys, N-acetyl-S-(2-carbamoyl-2-hydroxyethyl)-L-cysteine; HEMA, N-acetyl-S-(2-hydroxyethyl)-L-cysteine; HPMA, N-acetyl-S-(2-hydroxypropyl)-L-cysteine; 3-HPMA, N-acetyl-S-(3-hydroxypropyl)-L-cysteine; MA, mandelic acid; MHBMA, N-acetyl-S-(4-hydroxy-2-butenyl)-L-cysteine; PhEMA, N-acetyl-S-(phenyl-2-hydroxyethyl)-L-cysteine; PGA, phenylglyoxylic acid; PHEMA, N-acetyl-S-(phenyl)-L-cysteine; NAc-3HPM-Cys, N-acetyl-S-(3-hydroxypropyl-1-methyl)-L-cysteine; As, total arsenic; As(III), arsenous acid; AsB, arsenobetaine; AsC, arsenocholine; DMA, dimethylarsinic acid; MMA, monomethylarsonic acid; COT, total cotinine; HCOT, total hydroxycotinine; TNE-2, total nicotine equivalent-2.

## Data Availability

Publicly available datasets were analyzed in this study. The data from the National Health and Nutrition Examination Survey (NHANES) 2013–2014 and 2015–2016 cycles can be found here: https://wwwn.cdc.gov/nchs/nhanes/ (accessed on 26 May 2026).

## Supplementary Materials

The following supporting information can be downloaded at: https://www.mdpi.com/article/10.3390/toxics14060481/s1, Text S1: Study Population and Data Collection; Text S2: Laboratory Assessment of Environmental Toxicants; Text S3: Detailed Statistical Methodology; Figure S1: Flowchart of Sample Selection; Figure S2: Correlation of the Environmental Toxicants (58 Exposures); Figure S3: Sensitivity Analysis of Associations Between an Expanded Set of 65 Environmental Toxicants and Height-for-Age Z-Score (HAZ); Figure S4: Linear Dose–Response Relationship Between the Cumulative Weighted Quantile Sum (WQS) Index and Covariate-Adjusted HAZ; Figure S5: Nonlinear Exposure–Response Curves in the BKMR Model; Table S1: Characterization of Participants in This Study Before Data Interpolation; Table S2: Characteristics of 79 Environmental Contaminants or Metabolites of Environmental Contaminants; Table S3: Associations Between 63 Environmental Toxicants and Height-for-Age Z-Score in US Children and Adolescents (NHANES 2013–2016); Table S4: Weights of 23 Environmental Contaminants in the WQS Model; Table S5: Posterior Inclusion Probabilities (PIPs) of 47 Environmental Contaminants in the BKMR Model; Table S6: Adjusted Association Between Environmental Toxicants (58 Exposures) and HAZ in 1660 Children and Adolescents, Stratified by Age and Sex; Table S7: Mediation of Association Between Environmental Toxicants and HAZ by Sex Steroid Hormones (TT, E2, and SHBG) [24,32,33,34,35,72,73].
