# Supplementary material for: Environmental Toxicant Exposure and Height Among Children and Adolescents
Source: Toxics. 2026 May 30;14(6):481. doi: 10.3390/toxics14060481 (PMC13307051; doi:10.3390/toxics14060481)
Supplement: Supplementary file 1 [file toxics-14-00481-s001.zip › toxics-4299730-supplementary.pdf]

Supplementary Online Content

eMethods. Data Collection, Toxicant Detection, and Analysis

Text S1. Study Population and Data Collection

The National Health and Nutrition Examination Survey (NHANES) is a continuous, cross-sectional program designed to assess the health and nutritional status of the civilian, non-institutionalized population in the United States. Conducted by the National Center for Health Statistics (NCHS) at the Centers for Disease Control and Prevention (CDC), NHANES employs a complex, multistage, stratified, clustered probability sampling design. The sampling process proceeds hierarchically: selection of primary sampling units (PSUs, consisting of counties), followed by segments (clusters of households), specific households, and finally, eligible individuals within those households. Data collection integrates in-home interviews for demographic and health history information with standardized physical examinations and laboratory specimen collection conducted in Mobile Examination Centers (MECs). The MECs provide a controlled environment to ensure standardized conditions for anthropometric measurements and biospecimen collection across different geographic locations.[1]

For the current study, we utilized data from two survey cycles: 2013–2014 and 2015–2016. Our analysis was restricted to **Subsample A**, a designated subgroup of participants selected for comprehensive environmental biomonitoring. The NCHS Research Ethics Review Board approved the survey protocols, and written informed consent was obtained from all participants (or from parents/guardians for minors, with child assent where appropriate).

Text S2. Laboratory Assessment of Environmental Toxicants

Environmental toxicants were quantified in whole blood and urine specimens by the Division of Laboratory Sciences at the CDC's National Center for Environmental

Health, following standardized laboratory procedure manuals (LPMs)[2, 3]. A total of 58 toxicants were analyzed across 12 exposure families.

1. Blood Biomarkers (Hemoglobin Adducts)

Acrylamide, Glycidamide, and Ethylene Oxide (EO): Exposure to these compounds was assessed by measuring their specific hemoglobin (Hb) adducts. The analytical method involved a modified Edman reaction to cleave the N-terminal valine adducts from the globin chain. The resulting products were isolated and quantified using high-performance liquid chromatography coupled with tandem mass spectrometry (HPLC-MS/MS). Isotope-dilution techniques were employed to ensure high sensitivity and specificity.

Formaldehyde: Similar to the method described above, formaldehyde exposure was determined by measuring its Hb adducts using enzymatic digestion followed by HPLC-MS/MS analysis.

2. Urinary Biomarkers

Perchlorate, Nitrate, and Thiocyanate: These anions were simultaneously quantified using ion chromatography coupled with electrospray ionization tandem mass spectrometry (IC-ESI-MS/MS). Chromatographic separation was achieved using an IonPac AS16 column with a sodium hydroxide eluent, followed by detection in negative ion mode.

Metals (13 elements): Mass spectrometry can be used to directly measure multiple metals in urine samples after simple dilution. This method directly measures multiple metals in urine specimens using mass spectrometry after a simple dilution sample preparation step. Liquid samples are introduced into the mass spectrometer through the inductively coupled plasma (ICP) ionization source and reduced to small droplets in an argon aerosol via a nebulizer, after which the droplets enter the ICP. The ions first pass through a focusing region, followed by the dynamic reaction cell (DRC) and the quadrupole mass filter, and finally are selectively counted in rapid sequence at the detector,allowing individual isotopes of an element to be determined.

Arsenic and Arsenic Species: Speciated arsenic analysis differentiated between toxic inorganic forms and non-toxic organic forms. The method employed HPLC for species separation, followed by detection using ICP-DRC-MS.

Polycyclic Aromatic Hydrocarbons (PAHs): The urinary concentrations of PAH metabolites, specifically monohydroxylated PAHs (OH-PAHs), have been used as biomarkers of human exposure to select PAHs including naphthalene, fluorene, phenanthrene, and pyrene. Urine samples underwent enzymatic deconjugation (hydrolysis) to free metabolites from glucuronide and sulfate conjugates. Samples were then processed via on-line solid-phase extraction and analyzed using isotope-dilution HPLC-MS/MS.

Volatile Organic Compounds (VOCs) Metabolites: Exposure to VOCs was assessed by measuring specific urinary mercapturic acid metabolites. The analysis utilized ultra-performance liquid chromatography coupled with electrospray ionization tandem mass spectrometry (UPLC-ESI-MS/MS), allowing for high-throughput and sensitive quantification.

Nicotine Metabolites: "Total" urinary nicotine metabolites, including the free and glucuronide conjugated forms, are measured by two separate isotope dilution high performance liquid chromatography/tandem mass spectrometry (HPLC-MS/MS) methods based on a cotinine cut-off value of 20 ng/mL so that markedly different levels of nicotine exposure biomarkers can be optimally quantified in both users and nonusers.

Iodine: an essential element for thyroid function, is necessary for normal growth, development, and functioning of the brain and body. Iodine-deficiency disorder (IDD) is a well-documented global health problem that affects more than a billion people worldwide. The consequences of IDD include goiter, cretinism, intellectual impairment, brain damage, mental retardation, stillbirth, congenital deformities, and increased perinatal mortality. The detection methods and procedures for iodine are consistent with those for metals.

Text S3. Detailed Statistical Methodology

1. Data Processing and Toxicant Selection

Prior to statistical modeling, rigorous quality control was applied to the environmental monitoring data. Toxicants with distinct distributional properties were categorized based on detection frequencies: Biomarkers were excluded if they had excessive missing data (> 30%) or if the overall detection frequency was low (< 10%); biomarkers were included as binary variables if more than 30% of participants had a value below its detection limit, with these values set to zero; and in all other cases, the variable was natural logarithmically transformed. Continuous exposure variables were natural log-transformed (ln-transformed) to reduce skewness and approximate a normal distribution (details provided in **Table S1**).

2. Exposome-Wide Association Study (EWAS)

We fitted separate General Linear Models (GLMs) for each toxicant to estimate its independent association with Height-for-Age Z-score (HAZ). The basic model structure was:

$$Y_i = \beta_0 + \beta_1 X_i + \gamma^T \mathrm{C_i} + \epsilon_i$$

Where X_i represents the log-transformed concentration (or category) of the toxicant, and $\mathrm{C_i}$ represents the vector of covariates. To account for the multiple testing inherent in examining 58 toxicants, we applied the Benjamini-Hochberg (BH) procedure to control the False Discovery Rate (FDR) at 0.05.[4] When analyzing data of a complex survey, sampling weights are usually used to produce representative and unbiased statistics. However, it reduces the precision of the estimates, and even to some extent introduces over-adjustment bias if variables that were used to calculate the sampling weights were further adjusted in regression analyses. Thus, we presented our results without incorporating sampling weights, similar to some previous studies using NHANES data

3. Weighted Quantile Sum (WQS) Regression

WQS regression was employed to assess the cumulative effect of the toxicant mixture. This method constructs a weighted index (WQS_i) representing the mixture burden:

$$g(\mu) = \beta_0 + \beta_1 \left(\sum_{i=1}^{c} w_i q_i \right) + \beta^T \mathrm{C_i}$$

Where q_i is the quantile score (e.g., quartile or decile) of the i-th component, and w_i is the weight estimated through bootstrapping (2,000 iterations). The data were split into a training set (40%) for weight estimation and a validation set (60%) for hypothesis testing. Furthermore, based on the findings from our EWAS analysis, we applied a negative directional constraint to the overall mixture coefficient β to specifically evaluate the cumulative adverse effects of the toxicant mixture on height.[5]

4. Bayesian Kernel Machine Regression (BKMR)

To capture potential non-linear relationships and contribution without imposing parametric assumptions, we used BKMR with a Gaussian kernel. The model is specified as:

$$HAZ_i = h(z_{i1}, \dots, z_{iM}) + \beta^T \mathrm{C_i} + \epsilon_i$$

Where $h(z_{i1}, \dots, z_{iM})$ is an unknown flexible function of the mixture components z_i, and $\mathrm{C_i}$ represents covariates. We implemented the model with component-wise variable selection utilizing a spike-and-slab prior. The estimation was performed using 20,000 Markov Chain Monte Carlo (MCMC) iterations. We calculated and sort Posterior Inclusion Probabilities (PIPs) to identify influential toxicants. Univariate exposure-response functions were derived by fixing other mixture components at their median values. The cumulative effect was estimated by comparing the expected outcome when all exposures are at the 75th percentile versus the 25th percentile.[6]

5. Causal Mediation Analysis

To systematically explore the potential mediating role of sex steroid hormones (TT, E2, and SHBG) in the association between environmental toxicants and linear

growth, we performed a pairwise mediation analysis under the counterfactual framework.Let Y_i denote the outcome (HAZ), X_i denote the exposure (environmental toxicant), and M_i denote the mediator (sex steroid hormone). For each feasible combination of toxicant and hormone, we fitted two regression models:

(1) The mediator model regresses the mediator (M_i) on the toxicant (X_i) and the covariate vector ($\mathrm{C_i}$):

$$M_i = \alpha_0 + \alpha_1 X_i + \alpha_2^T \mathrm{C_i} + \epsilon_{i1}$$

(2) The outcome model regresses the outcome (Y_i) on the toxicant (X_i), the mediator (M_i), and the covariate vector ($\mathrm{C_i}$):

$$Y_i = \beta_0 + \beta_1 X_i + \beta_2 M_i + \beta_3^T \mathrm{C_i} + \epsilon_{i2}$$

In these equations, subscript i denotes the i-th participant. $\mathrm{C_i}$ represents the vector of covariates adjusted in the model. ϵ_{i1} and ϵ_{i2} are the error terms assumed to follow a normal distribution.Based on these models, we estimated the Average Causal Mediation Effect (ACME), representing the effect of the toxicant on HAZ mediated through sex hormones, and the Average Direct Effect (ADE), representing the effect of the toxicant on HAZ independent of the mediator. The ACME was calculated as the product of the coefficients（$\alpha_1 \times \beta_2$）, while the ADE corresponded to the coefficient β_1. The proportion mediated (PM) was calculated as:

$$\mathrm{PM} = \frac{NIE}{NIE + NDE} \times 100\%$$

To assess statistical significance, 95% confidence intervals (95%CI) for the indirect effects were obtained using nonparametric bootstrapping with 1,000 replications. The P-values for the ACME, ADE, and Total Effect were adjusted using the BH method to control the FDR at 5%.[7]

6. Covariates Definition

Models were adjusted for a set of prespecified covariates covering demographic, socioeconomic, and lifestyle factors. Demographic variables included age at examination (years), sex (male or female), and race/ethnicity (Mexican American, Other Hispanic, Non-Hispanic White, Non-Hispanic Black, or Other/Multi-racial).

Socioeconomic status was controlled for using the family poverty income ratio (PIR) as a continuous variable. Lifestyle covariates consisted of daily energy intake (kcal), calculated as the mean energy intake derived from two 24-hour dietary recall interviews, and the number of household smokers (categorical: 0, 1, 2, or 3). To address the incomplete matching of questionnaire modules across different age groups, physical activity frequency was defined as the maximum number of days per week reported for “moderate recreational activities,” “vigorous recreational activities,” or “being physically active for at least 60 minutes.”

Table S1. Characterization of Participants in This Study Before Data Interpolation

Characteristic		Overall	Male	Female
Age group [a]				
	6-11 years	786(47.3)	398(47.9)	388(46.8)
	12-19 years	874(52.7)	433(52.1)	441(53.2)
Age [b]		12.00[9.00,15.00]	12.00[9.00,15.00]	12.00[9.00,16.00]
Sex [a]				
Male		831(50.1)	831(100.0)	0(0.0)
Female		829(49.9)	0(0.0)	829(100.0)
Race [a]				
Mexican American		371(22.3)	166(20.0)	205(24.7)
Other Hispanic		197(11.9)	96(11.6)	101(12.2)
Non-Hispanic White		425(25.6)	230(27.7)	195(23.5)
Non-Hispanic Black		390(23.5)	201(24.2)	189(22.8)
Non-Hispanic Asian		160(9.6)	85(10.2)	75(9.0)
Other Race		117(7.0)	53(6.4)	64(7.7)
Poverty income ratio [b]		1.48[0.77,2.99]	1.50[0.84,3.23]	1.47[0.76,2.81]
Energy (kcal) [b]		1828.50 [1451.00,2286.50]	1997.50 [1619.62,2505.12]	1684.00 [1323.00,2076.00]
Physical activity [a]				
	0	44(2.7)	14(1.7)	30(3.6)
	1	81(4.9)	46(5.5)	35(4.2)
	2	121(7.3)	53(6.4)	68(8.2)
	3	151(9.1)	75(9.0)	76(9.2)
	4	116(7.0)	54(6.5)	62(7.5)
	5	292(17.6)	143(17.2)	149(18.0)
	6	88(5.3)	48(5.8)	40(4.8)
	7	607(36.6)	338(40.7)	269(32.4)
	NA	160(9.6)	60(7.2)	100(12.1)
Household smokers [a]				
	0	1221(73.6)	599(72.1)	622(75.0)
	1	266(16.0)	141(17.0)	125(15.1)
	2	114(6.9)	55(6.6)	59(7.1)
	3	26(1.6)	14(1.7)	12(1.4)
	NA	33(2.0)	22(2.6)	11(1.3)
Height (cm)[b]		152.40[133.80,164.20]	152.60[133.00,170.25]	152.30[134.90,160.70]
HAZ [b]		0.37[-0.46,1.10]	0.47[-0.34,1.19]	0.29[-0.51,1.04]
TT [b]		0.94[0.50,1.50]	1.51[0.44,1.93]	0.90[0.57,1.05]
E2 [b]		0.96[0.27,1.27]	0.62[0.27,1.07]	1.22[0.48,1.52]
SHBG [b]		3.92[3.43,4.43]	3.84[3.37,4.46]	4.00[3.49,4.41]

a Frequency and percentage [n (%)] are used to describe categorical variables.

b Median and interquartile range [median (Q1, Q3)] are used to describe variables with skewed distributions.

c Mean and standard deviation [mean (SD)] are used to describe continuous variables with a normal distribution.,

HAZ, Height-for-age Z-score; TT, total testosterone; E2, estradiol; SHBG, sex hormone –binding globulin.

Table S2. Characteristics of 79 Environmental Contaminants or Metabolites of Environmental Contaminants

Family	Exposure	Matrix	Unit	Mean±SD	Median (Q1,Q3)	LLOD	Below LLOD (%)	Missing (%)	Process
AMDGYD	Acrylamide	Serum	pmol/g Hb	47±31	41 (34, 51)	14.9	0	20	log e
	Glycideamide	Serum	pmol/g Hb	45±24	40 (31, 52)	12	0	20	log e
ETHOX	Ethylene Oxide	Serum	pmol/g Hb	27±34	20 (16, 28)	5.8	1	20	log e
FORMAL	Formaldehyde	Serum	nmol/g Hb	130±20	130 (120, 140)	54	0	20	log e
PAH	1-Hydroxynaphthalene	Urine	ng/L	2600±10000	860 (450, 1700)	42.4	0.4	7	log e
	2-Hydroxynaphthalene	Urine	ng/L	8300±10000	4800 (2300, 10000)	107	0	6	log e
	3-Hydroxyfluorene	Urine	ng/L	120±210	69 (38, 130)	5.7	1	6	log e
	2-Hydroxyfluorene	Urine	ng/L	240±390	150 (79, 270)	5.7	0.2	6	log e
	1-Hydroxyphenanthrene	Urine	ng/L	130±160	83 (47, 150)	6.4	1	6	log e
	1-Hydroxypyrene	Urine	ng/L	210±320	140 (76, 240)	49.5	20	6	log e
	2 & 3-Hydroxyphenanthrene	Urine	ng/L	170±220	110 (61, 200)	7.1	0.4	6	log e
PERNT	Perchlorate, urine	Urine	ng/mL	4.9±12	3.1 (1.6, 5.2)	0.117	0	7	log e
	Nitrate, urine	Urine	ng/mL	63000±57000	53000 (30000, 81000)	2990	0	7	log e
	Thiocyanate, urine	Urine	ng/mL	1400±1600	980 (500, 1700)	20.8	0	7	log e
UAS	Urinary Arsenous acid	Urine	ug/L	0.53±0.47	0.49 (0.08, 0.78)	0.08	30	6	log e
	Urinary Arsenic acid	Urine	ug/L	0.57±0.1	0.56 (0.56, 0.56)	0.56	100	6	Removed
	Urinary Arsenobetaine	Urine	ug/L	6.4±39	0.82 (0.82, 1.8)	0.82	70	6	2 categories
	Urinary Arsenocholine	Urine	ug/L	0.15±0.93	0.08 (0.08, 0.08)	0.08	90	6	2 categories
	Urinary Dimethylarsinic acid	Urine	ug/L	4.7±5.8	3.2 (1.9, 5.2)	1.35	20	6	log e
	Urinary Monomethylarsonic acid	Urine	ug/L	0.57±0.57	0.41 (0.14, 0.8)	0.14	40	6	2 categories
UCOT	Total Cotinine, urine	Urine	ng/mL	78±600	0.42 (0.13, 2.7)	0.021	3	9	log e
	Total Hydroxycotinine, urine	Urine	ng/mL	160±1200	0.72 (0.22, 4.9)	0.021	2	9	log e
	Anabasine, urine	Urine	ng/mL	2.2±4.9	0.36 (0.36, 1.1)	0.36	7	90	Removed

	Anatabine, urine	Urine	ng/mL	3±7.6	0.28 (0.28, 0.92)	0.28	7	90	Removed
	Cotinine-n-oxide, urine	Urine	ng/mL	110±240	13 (5.5, 60)	1.43	0.3	90	Removed
	Nicotine, urine	Urine	ng/mL	240±660	18 (7.4, 120)	7.42	4	90	Removed
	Nornicotine, urine	Urine	ng/mL	18±40	1.8 (1.8, 9.1)	1.77	6	90	Removed
	Nicotine-1 N-oxide, urine	Urine	ng/mL	83±210	9.3 (3.5, 52)	1.77	1	90	Removed
	TNE - 2	Urine	nmol/mL	1.3±9.4	0.0063 (0.002, 0.042)	2e-04	NA	9	log e
	TNE - 3	Urine	nmol/mL	14±30	1.7 (0.64, 6.8)	0.255	NA	90	Removed
	TNE - 6	Urine	nmol/mL	15±33	2 (0.72, 7.5)	0.2983	NA	90	Removed
	1-(3P)-1-but-4-carbox acid	Urine	ng/mL	180±400	27 (9.1, 95)	2.41	0	100	Removed
	TNE - 7	Urine	nmol/mL	19±42	2.8 (1, 12)	0.4318	NA	100	Removed
UHG	Urine Mercury	Urine	ng/mL	0.27±0.57	0.09 (0.09, 0.22)	0.09	60	5	2 categories
UIO	Iodine, urine	Urine	ng/mL	220±290	150 (82, 250)	10	0	5	log e
UM	Barium, urine	Urine	ug/L	2±2.9	1.2 (0.63, 2.4)	0.042	0.4	5	log e
	Cadmium, urine	Urine	ug/L	0.073±0.08	0.046 (0.025, 0.09)	0.025	40	5	2 categories
	Cobalt, urine	Urine	ug/L	0.74±2	0.57 (0.33, 0.88)	0.016	0.06	5	log e
	Cesium, urine	Urine	ug/L	5±3.1	4.5 (2.6, 6.7)	0.259	0	5	log e
	Molybdenum, urine	Urine	ug/L	73±61	58 (31, 97)	1.25	0	5	log e
	Manganese, urine	Urine	ug/L	0.16±0.26	0.092 (0.092, 0.16)	0.092	60	5	2 categories
	Lead, urine	Urine	ug/L	0.34±0.43	0.24 (0.14, 0.41)	0.02	1	5	log e
	Antimony, urine	Urine	ug/L	0.091±0.15	0.061 (0.033, 0.1)	0.016	10	5	log e
	Tin, urine	Urine	ug/L	1.5±2.8	0.62 (0.27, 1.5)	0.064	6	5	log e
	Strontium, urine	Urine	ug/L	110±98	89 (41, 160)	1.655	0.1	5	log e
	Thallium, urine	Urine	ug/L	0.2±0.13	0.18 (0.11, 0.27)	0.013	0.4	5	log e
	Tungsten, urine	Urine	ug/L	0.22±0.7	0.12 (0.057, 0.23)	0.013	5	5	log e
	Uranium, urine	Urine	ug/L	0.011±0.034	0.005 (0.0028, 0.01)	0.0014	20	5	log e
UTAS	Urinary arsenic, total	Urine	ug/L	12±41	5.3 (3, 9.5)	0.26	0	5	log e

	N-acel-S-(1,2-dichlorovinl)-L-cys	Urine	ng/mL	8.9±0.43	8.9 (8.9, 8.9)	8.9	100	10	Removed
	N-acel-S-(2,2-dichlorvinyl)-L-cys	Urine	ng/mL	6.4±3.2	3.3 (3.3, 9.8)	3.32	100	10	Removed
	2-methylhippuric acid	Urine	ng/mL	38±61	21 (9.4, 42)	3.54	10	10	log e
	3-methipurc acd & 4-methipurc acd	Urine	ng/mL	280±480	140 (72, 300)	5.66	0.7	7	log e
	N-ace-S-(2-carbamoylethyl)-L-cys	Urine	ng/mL	70±76	49 (26, 86)	2.31	0	9	log e
	N-ace-S-(N-methlcarbamoyl)-L-cys	Urine	ng/mL	92±120	63 (36, 110)	4.43	0.7	8	log e
	2-amnothiazolne-4-carbxylic acid	Urine	ng/mL	270±260	200 (91, 380)	10.6	2	9	log e
	N-acetyl-S-(benzyl)-L-cysteine	Urine	ng/mL	10±15	6.3 (3.3, 12)	0.354	0.7	7	log e
	N-acetyl-S-(n-propyl)-L-cysteine	Urine	ng/mL	6.4±12	2.4 (0.85, 6.6)	0.85	30	7	2 categories
	N-acetyl-S-(2-carbxyethyl)-L-cys	Urine	ng/mL	110±110	79 (41, 140)	4.92	1	7	log e
	N-acetyl-S-(2-cyanoethyl)-L-cyst	Urine	ng/mL	8.1±43	1.4 (0.7, 2.7)	0.354	20	8	log e
	N-ace-S-(3,4-dihidxybutl)-L-cys	Urine	ng/mL	380±280	310 (180, 510)	15.2	0	10	log e
UVOC	N-ace-S-(dimethylphenyl)-L-cys	Urine	ng/mL	0.35±0.011	0.35 (0.35, 0.35)	0.354	100	10	Removed
	N-ac-S-(2-carbmo-2-hydxel)-L-cys	Urine	ng/mL	11±8.5	6.6 (6.6, 13)	6.65	60	7	2 categories
	N-ace-S-(2-hydroxyethyl)-L-cys	Urine	ng/mL	1.5±2.7	0.89 (0.56, 1.7)	0.559	50	8	2 categories
	N-ace-S-(2-hydroxypropyl)-L-cys	Urine	ng/mL	51±200	26 (13, 46)	3.75	5	7	log e
	N-ace-S-(3-hydroxypropyl)-L-cys	Urine	ng/mL	310±320	210 (110, 390)	9.2	0.2	10	log e
	Mandelic acid	Urine	ng/mL	140±120	110 (63, 180)	8.5	2	9	log e
	N-A-S-(1-hydrxmet)-2-Prpn)-L-cys	Urine	ng/mL	0.5±0.027	0.5 (0.5, 0.5)	0.495	100	8	Removed
	N-Ac-S-(2-hydrxy-3-butenyl)-L-cys	Urine	ng/mL	0.53±0.34	0.5 (0.5, 0.5)	0.495	100	7	Removed
	N-A-S-(4-hydrxy-2-butenyl)-L-cys	Urine	ng/mL	6.6±8.7	4.5 (2.3, 8.1)	0.424	4	8	log e
	N-ace-S-(phenl-2-hydxyetl)-L-cys	Urine	ng/mL	1.1±2.6	0.5 (0.5, 1.1)	0.495	60	10	2 categories
	Phenylglyoxylic acid	Urine	ng/mL	220±170	180 (100, 300)	8.5	0.3	7	log e
	N-acetyl-S-(phenyl)-L-cysteine	Urine	ng/mL	1±1.3	0.42 (0.42, 1.2)	0.424	50	8	2 categories
	N-A-S-(3-hydrxprpl-1-metl)-L-cys	Urine	ng/mL	270±430	190 (100, 320)	7.97	0	7	log e
	N-acetyl-S-(trichlorovinyl)-L-cys	Urine	ng/mL	2.1±0	2.1 (2.1, 2.1)	2.12	100	7	Removed

2-thoxothazlidne-4-carbxylic acid	Urine	ng/mL	27±81	7.9 (7.9, 14)	7.9	70	50	Removed
CYHA cysteine	Urine	ng/mL	2.7±6.3	1.8 (1.8, 1.8)	1.84	90	60	Removed
IPM1 cysteine	Urine	ng/mL	3.2±2.8	2.3 (1, 4)	1.01	30	60	Removed
IPM3 cysteine	Urine	ng/mL	4.9±7.4	2.8 (1.3, 5.7)	0.85	20	60	Removed

Abbreviations: AMDGYD, acrylamide and glycidamide; ETHOX, ethylene oxide; FORMAL, formaldehyde; UIO, iodine; PERNT, perchlorate, nitrate, and thiocyanate; UM, metals; PAH, polycyclic aromatic hydrocarbons; UHG, mercury; UVOC, volatile organic compounds; UTAS, total arsenic; UAS, speciated arsenics; UCOT, cotinine.

Table S3. Associations Between 63 Environmental Toxicants and Height-for-Age Z-Score in US Children and Adolescents (NHANES 2013-2016)

Exposure family by toxicant exposure	Median (Q1,Q3)	process	Model 1[a]		Model 2[b]	
			β（95%CI）	P value[c]	β（95%CI）	P value[c]
UVOC						
1,2-DCVC	NA	2 categories	0.774 (-1.491, 3.038)	0.67	0.011 (-2.118, 2.140)	0.99
2-MHA	20.900 (9.440–42.500)	log e	-0.037 (-0.109, 0.036)	0.57	-0.047 (-0.116, 0.021)	0.35
3-MHA/4-MHA	142.000 (71.600–297.500)	log e	-0.006 (-0.076, 0.065)	0.92	-0.035 (-0.101, 0.032)	0.49
NAc2CE-Cys	49.400 (26.500–86.450)	log e	0.026 (-0.041, 0.094)	0.63	-0.023 (-0.087, 0.041)	0.63
NAcMC-Cys	63.250 (35.800–106.000)	log e	-0.026 (-0.088, 0.036)	0.61	-0.042 (-0.102, 0.018)	0.34
2-ATCA	203.500 (90.775–379.750)	log e	0.092 (0.021, 0.164)	0.12	-0.208 (-0.292, -0.125)	<0.001
BMA	6.260 (3.262–11.700)	log e	0.060 (-0.009, 0.128)	0.33	-0.086 (-0.155, -0.018)	0.042
NAcSP-Cys	NA	2 categories	-0.025 (-0.145, 0.096)	0.82	0.043 (-0.071, 0.158)	0.61
CEMA	79.400 (41.250–141.500)	log e	0.119 (0.048, 0.189)	0.015	-0.011 (-0.079, 0.057)	0.8
CYMA	1.380 (0.699–2.670)	log e	-0.041 (-0.094, 0.012)	0.36	-0.056 (-0.108, -0.005)	0.085
DHBMA	314.000 (176.000–507.000)	log e	0.115 (0.050, 0.180)	0.011	-0.042 (-0.109, 0.026)	0.41
DPMA	NA	2 categories	-0.629 (-2.231, 0.973)	0.63	-0.565 (-2.066, 0.937)	0.61
NAc-2CHE-Cys	NA	2 categories	0.077 (-0.036, 0.189)	0.42	0.078 (-0.029, 0.184)	0.32
HEMA	NA	2 categories	0.120 (0.009, 0.232)	0.2	0.045 (-0.061, 0.150)	0.58
HPMA	25.500 (13.100–45.875)	log e	-0.010 (-0.071, 0.050)	0.82	-0.084 (-0.142, -0.026)	0.021
3-HPMA	210.000 (109.000–394.000)	log e	0.045 (-0.023, 0.112)	0.42	-0.028 (-0.094, 0.037)	0.58
MA	113.000 (62.925–185.000)	log e	0.043 (-0.020, 0.105)	0.42	-0.053 (-0.114, 0.008)	0.22
MHBMA1	NA	2 categories	0.742 (-0.859, 2.344)	0.57	0.388 (-1.115, 1.891)	0.7
MHBMA2	NA	2 categories	-0.175 (-0.533, 0.183)	0.57	0.032 (-0.309, 0.373)	0.88
MHBMA	4.460 (2.260–8.100)	log e	0.034 (-0.033, 0.101)	0.57	-0.082 (-0.147, -0.018)	0.042
PhEMA	NA	2 categories	0.098 (-0.015, 0.212)	0.33	0.054 (-0.053, 0.160)	0.5
PGA	182.000 (104.000–295.250)	log e	0.113 (0.051, 0.175)	0.011	-0.019 (-0.082, 0.044)	0.68
PHEMA	NA	2 categories	0.066 (-0.045, 0.178)	0.47	0.026 (-0.079, 0.131)	0.71
NAc-3HPM-Cys	187.500 (105.000–316.000)	log e	0.020 (-0.036, 0.075)	0.67	-0.086 (-0.142, -0.031)	0.014
UTAS						
As	5.295 (3.012–9.498)	log e	0.020 (-0.047, 0.086)	0.72	-0.019 (-0.086, 0.048)	0.68
UM						
Ba	1.250 (0.630–2.360)	log e	0.048 (-0.023, 0.120)	0.42	-0.010 (-0.080, 0.061)	0.83
Cd	NA	2 categories	-0.054 (-0.167, 0.060)	0.57	0.142 (0.030, 0.255)	0.042
Co	0.567 (0.327–0.876)	log e	0.049 (-0.021, 0.119)	0.42	-0.053 (-0.124, 0.018)	0.32
Cs	4.526 (2.643–6.728)	log e	0.092 (0.016, 0.168)	0.13	-0.170 (-0.262, -0.079)	0.0022

Mo	57.965 (30.685–96.675)	log e	0.044 (-0.027, 0.115)	0.45	-0.173 (-0.248, -0.097)	<0.001
Mn	NA	2 categories	0.093 (-0.024, 0.209)	0.36	0.063 (-0.047, 0.173)	0.44
Pb	0.240 (0.140–0.410)	log e	0.023 (-0.049, 0.094)	0.7	-0.243 (-0.321, -0.165)	<0.001
Sb	0.061 (0.033–0.105)	log e	0.029 (-0.040, 0.099)	0.61	-0.171 (-0.244, -0.099)	<0.001
Sn	0.620 (0.270–1.527)	log e	0.050 (-0.030, 0.130)	0.45	-0.289 (-0.378, -0.201)	<0.001
Sr	89.215 (41.278–158.535)	log e	-0.110 (-0.181, -0.040)	0.029	-0.113 (-0.182, -0.044)	0.0083
Tl	0.176 (0.107–0.269)	log e	0.068 (-0.008, 0.144)	0.33	-0.090 (-0.173, -0.008)	0.085
W	0.119 (0.057–0.235)	log e	0.084 (0.014, 0.153)	0.13	-0.057 (-0.127, 0.012)	0.25
U	0.005 (0.003–0.010)	log e	-0.014 (-0.082, 0.054)	0.82	-0.033 (-0.099, 0.033)	0.5
UIO						
I	148.000 (82.400–249.850)	log e	0.146 (0.072, 0.220)	0.0076	-0.062 (-0.142, 0.018)	0.29
UHG						
Hg	NA	2 categories	0.087 (-0.026, 0.200)	0.36	0.185 (0.078, 0.293)	0.0052
UCOT						
COT	0.418 (0.126–2.705)	log e	-0.009 (-0.078, 0.060)	0.86	-0.050 (-0.127, 0.028)	0.39
HCOT	0.719 (0.218–4.945)	log e	0.012 (-0.058, 0.081)	0.82	-0.050 (-0.129, 0.028)	0.39
TNE-2	0.006 (0.002–0.042)	NA	-0.001 (-0.001, -0.000)	0.2	-0.000 (-0.001, 0.000)	0.68
UAS						
As(III)	0.490 (0.080–0.780)	log e	-0.020 (-0.096, 0.055)	0.74	-0.040 (-0.113, 0.032)	0.45
AsV	NA	2 categories	-0.095 (-0.449, 0.259)	0.74	-0.135 (-0.466, 0.197)	0.6
AsB	NA	2 categories	0.073 (-0.048, 0.194)	0.47	0.134 (0.019, 0.250)	0.066
AsC	NA	2 categories	0.006 (-0.157, 0.169)	0.96	0.071 (-0.082, 0.224)	0.55
DMA	3.210 (1.920–5.210)	log e	-0.010 (-0.078, 0.059)	0.85	-0.098 (-0.170, -0.025)	0.036
MMA	NA	2 categories	-0.099 (-0.215, 0.017)	0.33	-0.003 (-0.114, 0.109)	0.98
PERNT						
ClO4-	3.080 (1.620–5.190)	log e	0.084 (0.017, 0.151)	0.13	-0.074 (-0.145, -0.004)	0.099
NO3-	53000.000 (30450.000–80850.000)	log e	0.032 (-0.036, 0.100)	0.57	-0.148 (-0.222, -0.074)	<0.001
SCN-	982.000 (500.250–1660.000)	log e	0.064 (-0.006, 0.134)	0.32	-0.013 (-0.081, 0.054)	0.77
PAH						
1-OHN	859.000 (449.000–1700.000)	log e	-0.012 (-0.075, 0.051)	0.82	-0.083 (-0.143, -0.023)	0.029
2-OHN	4770.000 (2293.000–10180.000)	log e	-0.004 (-0.079, 0.072)	0.96	0.014 (-0.060, 0.088)	0.78
3-OHFlu	69.000 (38.000–128.000)	log e	0.012 (-0.055, 0.079)	0.82	-0.083 (-0.147, -0.019)	0.042
2-OHFlu	147.000 (79.000–270.000)	log e	0.050 (-0.015, 0.114)	0.36	-0.019 (-0.081, 0.043)	0.68
1-OHPhe	83.000 (47.000–154.000)	log e	0.065 (-0.002, 0.132)	0.27	-0.018 (-0.084, 0.047)	0.68
1-OHP	137.000 (76.000–235.000)	log e	0.026 (-0.042, 0.095)	0.63	-0.103 (-0.172, -0.034)	0.018
2,3-OHPhe	113.000	log e	0.072 (0.004, 0.141)	0.2	-0.040 (-0.107, 0.027)	0.42

		(61.000–197.000)					
FORMAL							
	HCHO	131.000 (121.000–143.000)	log e	0.001 (-0.058, 0.061)	0.96	0.022 (-0.034, 0.078)	0.6
ETHOX							
	EO	20.400 (16.270–27.910)	log e	-0.038 (-0.093, 0.018)	0.42	-0.065 (-0.119, -0.012)	0.052
AMDGYD							
	ACR	41.400 (33.900–50.900)	log e	-0.050 (-0.111, 0.011)	0.35	-0.074 (-0.131, -0.016)	0.042
	GLY	39.900 (31.000–52.000)	log e	-0.034 (-0.103, 0.036)	0.57	-0.141 (-0.209, -0.074)	<0.001

Note:

a Unadjusted model.

b Constructed by adjusting for age, sex, race/ethnicity, daily energy intake, weekly physical activity frequency, numbers of family smokers and family income-to-poverty ratio.

c. Adjusted to control the false discovery rate at 5%.

Abbreviations: AMDGYD, acrylamide and glycidamide; ETHOX, ethylene oxide; FORMAL, formaldehyde; UIO, iodine; PERNT, perchlorate, nitrate, and thiocyanate; UM, metals; PAH, polycyclic aromatic hydrocarbons; UHG, mercury; UVOC, volatile organic compounds; UTAS, total arsenic; UAS, speciated arsenics; UCOT, cotinine. ACR, acrylamide; GLY, glycidamide; EO, ethylene oxide; HCHO, formaldehyde; I, iodine; ClO_4^-, perchlorate; NO_3^-, nitrate; SCN^-, thiocyanate; Ba, barium; Cd, cadmium; Co, cobalt; Cs, cesium; Mo, molybdenum; Mn, manganese; Pb, lead; Sb, antimony; Sn, tin; Sr, strontium; Tl, thallium; W, tungsten; U, uranium; 1-OHN, 1-hydroxynaphthalene; 2-OHN, 2-hydroxynaphthalene; 3-OHFlu, 3-hydroxyfluorene; 2-OHFlu, 2-hydroxyfluorene; 1-OHPhe, 1-hydroxyphenanthrene; 2,3-OHPhe, 2- and 3-hydroxyphenanthrene; 1-OHP, 1-hydroxypyrene; Hg, mercury; 2-MHA, 2-methylhippuric acid; 3-MHA/4-MHA, 3-methylhippuric acid and 4-methylhippuric acid; NAc2CE-Cys, N-acetyl-S-(2-carbamoylethyl)-L-cysteine; NAcMC-Cys, N-acetyl-S-(N-methylcarbamoyl)-L-cysteine; 2-ATCA, 2-aminothiazoline-4-carboxylic acid; BMA, N-acetyl-S-(benzyl)-L-cysteine; NAcSP-Cys, N-acetyl-S-(n-propyl)-L-cysteine; CEMA, N-acetyl-S-(2-carboxyethyl)-L-cysteine; CYMA, N-acetyl-S-(2-cyanoethyl)-L-cysteine; DHBMA, N-acetyl-S-(3,4-dihydroxybutyl)-L-cysteine; NAc-2CHE-Cys, N-acetyl-S-(2-carbamoyl-2-hydroxyethyl)-L-cysteine; HEMA, N-acetyl-S-(2-hydroxyethyl)-L-cysteine; HPMA, N-acetyl-S-(2-hydroxypropyl)-L-cysteine; 3-HPMA, N-acetyl-S-(3-hydroxypropyl)-L-cysteine; MA, mandelic acid; MHBMA, N-acetyl-S-(4-hydroxy-2-butenyl)-L-cysteine; PhEMA, N-acetyl-S-(phenyl-2-hydroxyethyl)-L-cysteine; PGA, phenylglyoxylic acid; PHEMA, N-acetyl-S-(phenyl)-L-cysteine; NAc-3HPM-Cys, N-acetyl-S-(3-hydroxypropyl-1-methyl)-L-cysteine; As, total arsenic; As(III), arsenous acid; AsB, arsenobetaine; AsC, arsenocholine; DMA, dimethylarsinic acid; MMA, monomethylarsonic acid; COT, total cotinine; HCOT, total hydroxycotinine; TNE-2, total nicotine equivalent-2.

Table S4. Weights of 23 Environmental Contaminants in WQS model.

Exposure	Abbreviation	Weights
2-amnothiazolne-4-carbxylic acid	2-ATCA	0.178
Ethylene Oxide	EO	0.159
Tin, urine	Sn	0.134
Nitrate, urine	NO3-	0.094
Lead, urine	Pb	0.085
N-acetyl-S-(benzyl)-L-cysteine	BMA	0.058
Glycideamide	GLY	0.052
Strontium, urine	Sr	0.039
Acrylamide	ACR	0.032
Antimony, urine	Sb	0.029
1-Hydroxypyrene	1-OHP	0.027
N-ace-S-(2-hydroxypropyl)-L-cys	HPMA	0.025
Perchlorate, urine	ClO4-	0.016
Urinary Dimethylarsinic acid	DMA	0.016
Molybdenum, urine	Mo	0.013
N-A-S-(3-hydrxprpl-1-metl)-L-cys	NAc-3HPM-Cys	0.013
Cesium, urine	Cs	0.013
1-Hydroxynaphthalene	1-OHN	0.010
3-Hydroxyfluorene	3-OHFlu	0.005
N-A-S-(4-hydrxy-2-butenyl)-L-cys	MHBMA	0.001
N-acetyl-S-(2-cyanoethyl)-L-cyst	CYMA	0.001

Table S5. Posterior inclusion probabilities (PIPs) of 47 Environmental Contaminants in BKMR model.

Family	label	Abbreviation	PIP
UM	Tin, urine	Sn	1
UVOC	2-amnothiazolne-4-carbxylic acid	2-ATCA	1
PERNT	Nitrate, urine	NO3-	0.9874
UVOC	N-acetyl-S-(2-carbxyethyl)-L-cys	CEMA	0.9804
ETHOX	Ethylene Oxide	EO	0.9745
UM	Antimony, urine	Sb	0.9646
UM	Cobalt, urine	Co	0.9465
PAH	2-Hydroxynaphthalene	2-OHN	0.9463
UM	Lead, urine	Pb	0.9359
AMDGYD	Glycideamide	GLY	0.9333
UM	Molybdenum, urine	Mo	0.9291
UIO	Iodine, urine	I	0.9189
UVOC	2-methylhippuric acid	2-MHA	0.9132
UVOC	N-A-S-(3-hydrxprpl-1-metl)-L-cys	NAc-3HPM-Cys	0.9127
PERNT	Thiocyanate, urine	SCN-	0.8937
UVOC	N-acetyl-S-(benzyl)-L-cysteine	BMA	0.8825
UM	Barium, urine	Ba	0.8815
UVOC	Phenylglyoxylic acid	PGA	0.8554
UCOT	Total Cotinine, urine	COT	0.8465
UCOT	Total nicotine equivalent-2	TNE-2	0.844
PAH	3-Hydroxyfluorene	3-OHFlu	0.8374
PAH	2 & 3-Hydroxyphenanthrene	2,3-OHPhe	0.8299
UVOC	N-acetyl-S-(2-cyanoethyl)-L-cyst	CYMA	0.8268
UVOC	N-ace-S-(3,4-dihidxybutl)-L-cys	DHBMA	0.8255
PERNT	Perchlorate, urine	ClO4-	0.8227
UM	Thallium, urine	Tl	0.8216

PAH	1-Hydroxypyrene	1-OHP	0.8211
UTAS	Urinary arsenic, total	As	0.8211
PAH	2-Hydroxyfluorene	2-OHFlu	0.8137
PAH	1-Hydroxynaphthalene	1-OHN	0.8115
UM	Uranium, urine	U	0.8058
UAS	Urinary Arsenous acid	As(III)	0.8024
UVOC	N-ace-S-(3-hydroxypropyl)-L-cys	3-HPMA	0.7971
UM	Tungsten, urine	W	0.7911
UM	Strontium, urine	Sr	0.791
UM	Cesium, urine	Cs	0.7891
PAH	1-Hydroxyphenanthrene	1-OHPhe	0.7884
UAS	Urinary Dimethylarsinic acid	DMA	0.7706
AMDGYD	Acrylamide	ACR	0.7644
UCOT	Total Hydroxycotinine, urine	HCOT	0.764
FORMAL	Formaldehyde	HCHO	0.7614
UVOC	N-ace-S-(2-carbamoylethyl)-L-cys	NAc2CE-Cys	0.7593
UVOC	3-methipurc acd & 4-methipurc acd	3-MHA/4-MHA	0.7459
UVOC	Mandelic acid	MA	0.723
UVOC	N-ace-S-(2-hydroxypropyl)-L-cys	HPMA	0.7173
UVOC	N-A-S-(4-hydrxy-2-butenyl)-L-cys	MHBMA	0.7127
UVOC	N-ace-S-(N-methlcarbamoyl)-L-cys	NAcMC-Cys	0.6601

Table S6. Adjusted Association Between Environmental Toxicants (58 Exposures) and HAZ in 1660 children and adolescents (EWAS Analysis) stratified by age and sex.

Family	Exposure	6-11 years		12-19 years		Male		Female	
		β (95%CI)	p value [a]	β (95%CI)	p value [a]	β (95%CI)	p value [a]	β (95%CI)	p value [a]
AMDGYD	ACR	-0.02 (-0.14, 0.09)	0.75	-0.04 (-0.10, 0.03)	0.73	-0.11 (-0.19, -0.03)	0.02	-0.01 (-0.10, 0.07)	0.88
AMDGYD	GLY	-0.06 (-0.19, 0.07)	0.62	-0.08 (-0.15, -0.00)	0.3	-0.13 (-0.22, -0.04)	0.01	-0.08 (-0.18, 0.01)	0.49
ETHOX	EO	-0.09 (-0.19, 0.02)	0.29	-0.03 (-0.09, 0.03)	0.8	-0.13 (-0.20, -0.05)	0.01	-0.02 (-0.10, 0.06)	0.88
FORMAL	HCHO	0.06 (-0.02, 0.15)	0.34	-0.02 (-0.10, 0.05)	0.85	0.04 (-0.05, 0.12)	0.5	-0.00 (-0.08, 0.07)	0.96
UIO	I	-0.19 (-0.31, -0.08)	0.01	0.06 (-0.05, 0.16)	0.76	-0.05 (-0.16, 0.07)	0.5	-0.08 (-0.19, 0.03)	0.57
PERNT	ClO4-	-0.15 (-0.24, -0.05)	0.03	0.04 (-0.06, 0.13)	0.8	-0.10 (-0.20, -0.01)	0.07	-0.05 (-0.15, 0.05)	0.7
PERNT	NO3-	-0.22 (-0.33, -0.11)	0	-0.06 (-0.15, 0.04)	0.73	-0.22 (-0.32, -0.11)	0	-0.09 (-0.19, 0.01)	0.49
PERNT	SCN-	-0.07 (-0.17, 0.04)	0.43	0.06 (-0.02, 0.15)	0.71	-0.04 (-0.14, 0.06)	0.53	0.02 (-0.08, 0.11)	0.88
UM	Ba	-0.02 (-0.13, 0.08)	0.75	-0.03 (-0.13, 0.06)	0.8	-0.06 (-0.16, 0.04)	0.39	0.01 (-0.09, 0.10)	0.93
UM	Cd	0.01 (-0.15, 0.17)	0.88	0.21 (0.06, 0.37)	0.06	0.19 (0.03, 0.36)	0.05	0.12 (-0.04, 0.27)	0.56
UM	Co	-0.14 (-0.27, -0.02)	0.09	0.02 (-0.06, 0.11)	0.9	-0.05 (-0.15, 0.06)	0.5	-0.01 (-0.11, 0.08)	0.88
UM	Cs	-0.30 (-0.44, -0.16)	<0.001	-0.02 (-0.14, 0.10)	0.99	-0.25 (-0.39, -0.11)	0	-0.11 (-0.23, 0.01)	0.49
UM	Mo	-0.24 (-0.35, -0.13)	<0.001	-0.07 (-0.16, 0.03)	0.73	-0.15 (-0.26, -0.04)	0.02	-0.17 (-0.27, -0.06)	0.04
UM	Mn	0.06 (-0.10, 0.22)	0.71	0.02 (-0.13, 0.16)	0.99	0.06 (-0.10, 0.22)	0.53	0.04 (-0.10, 0.19)	0.85
UM	Pb	-0.23 (-0.35, -0.12)	0	-0.18 (-0.28, -0.08)	0.02	-0.34 (-0.46, -0.23)	<0.001	-0.11 (-0.22, -0.01)	0.41
UM	Sb	-0.16 (-0.28, -0.05)	0.04	-0.15 (-0.24, -0.06)	0.02	-0.24 (-0.35, -0.14)	<0.001	-0.08 (-0.18, 0.01)	0.49
UM	Sn	-0.29 (-0.43, -0.16)	<0.001	-0.19 (-0.30, -0.07)	0.02	-0.31 (-0.44, -0.19)	<0.001	-0.20 (-0.33, -0.07)	0.04
UM	Sr	-0.11 (-0.20, -0.01)	0.13	-0.07 (-0.17, 0.02)	0.71	-0.14 (-0.24, -0.04)	0.02	-0.09 (-0.18, 0.01)	0.49
UM	Tl	-0.13 (-0.25, -0.01)	0.13	0.02 (-0.09, 0.13)	0.99	-0.15 (-0.27, -0.02)	0.04	0.02 (-0.09, 0.13)	0.88
UM	W	-0.11 (-0.22, -0.01)	0.13	0.00 (-0.09, 0.09)	0.99	-0.04 (-0.14, 0.06)	0.53	-0.07 (-0.16, 0.02)	0.56
UM	U	-0.11 (-0.21, -0.00)	0.14	0.02 (-0.06, 0.11)	0.87	-0.06 (-0.15, 0.04)	0.39	-0.02 (-0.11, 0.07)	0.88

PAH	1-OHN	-0.07 (-0.16, 0.02)	0.34	-0.05 (-0.13, 0.03)	0.73	-0.14 (-0.23, -0.05)	0.01	-0.04 (-0.12, 0.04)	0.7
PAH	2-OHN	0.10 (-0.01, 0.21)	0.2	-0.00 (-0.10, 0.10)	0.99	0.06 (-0.05, 0.17)	0.44	0.00 (-0.10, 0.10)	0.96
PAH	3-OHFlu	-0.06 (-0.16, 0.05)	0.52	-0.02 (-0.10, 0.06)	0.87	-0.09 (-0.18, 0.00)	0.1	-0.05 (-0.14, 0.04)	0.7
PAH	2-OHFlu	0.04 (-0.06, 0.14)	0.66	-0.01 (-0.08, 0.07)	0.99	-0.04 (-0.13, 0.05)	0.5	0.01 (-0.08, 0.09)	0.93
PAH	1-OHPhe	0.04 (-0.05, 0.14)	0.66	-0.03 (-0.12, 0.05)	0.8	-0.05 (-0.15, 0.04)	0.4	0.03 (-0.06, 0.12)	0.85
PAH	1-OHP	-0.02 (-0.13, 0.08)	0.75	-0.12 (-0.21, -0.03)	0.11	-0.15 (-0.25, -0.05)	0.01	-0.03 (-0.13, 0.08)	0.88
PAH	2,3-OHPhe	0.02 (-0.07, 0.12)	0.75	-0.03 (-0.12, 0.06)	0.8	-0.07 (-0.16, 0.03)	0.28	0.01 (-0.08, 0.11)	0.9
UHG	Hg	0.13 (-0.03, 0.29)	0.29	0.19 (0.06, 0.33)	0.06	0.26 (0.11, 0.41)	0.01	0.08 (-0.07, 0.23)	0.7
UVOC	2-MHA	0.05 (-0.06, 0.16)	0.65	-0.03 (-0.12, 0.05)	0.8	-0.02 (-0.12, 0.08)	0.74	-0.06 (-0.15, 0.04)	0.68
UVOC	3-MHA/4-MHA	0.10 (-0.01, 0.21)	0.21	-0.04 (-0.12, 0.04)	0.76	-0.01 (-0.10, 0.09)	0.93	-0.04 (-0.13, 0.05)	0.83
UVOC	NAc2CE-Cys	-0.03 (-0.14, 0.07)	0.75	0.00 (-0.08, 0.08)	0.99	-0.08 (-0.17, 0.01)	0.16	0.02 (-0.07, 0.11)	0.88
UVOC	NAcMC-Cys	0.03 (-0.08, 0.13)	0.75	-0.04 (-0.11, 0.04)	0.76	-0.14 (-0.23, -0.05)	0.01	0.06 (-0.02, 0.14)	0.56
UVOC	2-ATCA	-0.22 (-0.37, -0.07)	0.03	-0.17 (-0.26, -0.07)	0.02	-0.21 (-0.33, -0.09)	0	-0.21 (-0.34, -0.09)	0.04
UVOC	BMA	-0.10 (-0.20, -0.00)	0.16	-0.05 (-0.14, 0.04)	0.76	-0.12 (-0.22, -0.03)	0.03	-0.06 (-0.16, 0.03)	0.62
UVOC	NAcSP-Cys	0.05 (-0.12, 0.21)	0.75	-0.12 (-0.27, 0.04)	0.71	0.08 (-0.09, 0.25)	0.5	-0.11 (-0.27, 0.04)	0.56
UVOC	CEMA	0.04 (-0.07, 0.14)	0.7	-0.03 (-0.12, 0.05)	0.8	-0.06 (-0.15, 0.04)	0.4	0.03 (-0.06, 0.12)	0.85
UVOC	CYMA	-0.01 (-0.12, 0.10)	0.88	-0.02 (-0.08, 0.03)	0.8	-0.14 (-0.21, -0.06)	0	0.04 (-0.04, 0.11)	0.7
UVOC	DHBMA	-0.09 (-0.19, 0.01)	0.23	0.01 (-0.08, 0.10)	0.99	-0.12 (-0.22, -0.02)	0.04	0.01 (-0.08, 0.10)	0.93
UVOC	NAc-2CHE-Cys	0.06 (-0.09, 0.22)	0.69	0.05 (-0.09, 0.18)	0.8	0.07 (-0.09, 0.22)	0.5	0.05 (-0.10, 0.19)	0.85
UVOC	HEMA	0.06 (-0.10, 0.22)	0.7	-0.01 (-0.15, 0.13)	0.99	-0.04 (-0.20, 0.11)	0.64	0.11 (-0.04, 0.25)	0.56
UVOC	HPMA	-0.11 (-0.20, -0.02)	0.08	-0.03 (-0.10, 0.05)	0.8	-0.16 (-0.25, -0.08)	0	-0.03 (-0.11, 0.05)	0.81
UVOC	3-HPMA	-0.02 (-0.12, 0.08)	0.75	-0.04 (-0.12, 0.04)	0.79	-0.14 (-0.24, -0.04)	0.01	0.03 (-0.05, 0.12)	0.83
UVOC	MA	-0.02 (-0.11, 0.07)	0.75	-0.04 (-0.13, 0.04)	0.76	-0.10 (-0.19, -0.01)	0.06	-0.03 (-0.11, 0.06)	0.85
UVOC	MHBMA	-0.06 (-0.16, 0.04)	0.43	-0.05 (-0.13, 0.03)	0.73	-0.15 (-0.24, -0.05)	0.01	-0.02 (-0.11, 0.07)	0.88
UVOC	PhEMA	0.03 (-0.13, 0.18)	0.78	0.08 (-0.06, 0.21)	0.76	0.07 (-0.08, 0.23)	0.5	0.03 (-0.11, 0.17)	0.88
UVOC	PGA	0.06 (-0.03, 0.15)	0.42	-0.06 (-0.14, 0.02)	0.71	-0.07 (-0.16, 0.02)	0.21	0.04 (-0.05, 0.12)	0.74

UVOC	PHEMA	0.04 (-0.11, 0.20)	0.75	0.00 (-0.13, 0.14)	0.99	-0.01 (-0.16, 0.15)	0.93	0.05 (-0.10, 0.19)	0.85
UVOC	NAc-3HPM-Cys	-0.13 (-0.22, -0.05)	0.01	-0.00 (-0.08, 0.07)	0.99	-0.20 (-0.28, -0.12)	<0.001	-0.01 (-0.08, 0.07)	0.93
UTAS	As	-0.02 (-0.12, 0.09)	0.78	0.00 (-0.08, 0.08)	0.99	0.01 (-0.09, 0.11)	0.88	-0.04 (-0.13, 0.04)	0.7
UAS	As(III)	-0.04 (-0.14, 0.07)	0.71	-0.00 (-0.10, 0.09)	0.99	-0.04 (-0.14, 0.07)	0.58	-0.02 (-0.12, 0.08)	0.88
UAS	AsB	0.22 (0.04, 0.40)	0.08	0.11 (-0.04, 0.25)	0.71	0.18 (0.02, 0.35)	0.07	0.10 (-0.06, 0.26)	0.68
UAS	AsC	-0.04 (-0.28, 0.20)	0.81	0.06 (-0.12, 0.25)	0.8	0.04 (-0.18, 0.25)	0.79	0.04 (-0.17, 0.26)	0.88
UAS	DMA	-0.07 (-0.18, 0.04)	0.43	-0.07 (-0.16, 0.02)	0.71	-0.13 (-0.24, -0.02)	0.04	-0.05 (-0.15, 0.05)	0.7
UAS	MMA	-0.04 (-0.20, 0.12)	0.75	0.01 (-0.14, 0.17)	0.99	0.01 (-0.15, 0.18)	0.9	-0.02 (-0.17, 0.13)	0.88
UCOT	COT	0.01 (-0.14, 0.16)	0.88	0.01 (-0.08, 0.10)	0.99	-0.17 (-0.28, -0.06)	0.01	0.13 (0.01, 0.25)	0.41
UCOT	HCOT	-0.04 (-0.19, 0.11)	0.75	0.01 (-0.08, 0.10)	0.99	-0.18 (-0.29, -0.07)	0.01	0.10 (-0.01, 0.21)	0.49
UCOT	TNE-2	0.00 (-0.00, 0.01)	0.34	0.00 (-0.00, 0.00)	0.99	-0.00 (-0.00, 0.00)	0.21	0.00 (-0.00, 0.00)	0.7

Abbreviations: CI, confidence interval; ACME, average causal mediation effect; ADE, average direct effect; TE, total effect;AMDGYD, acrylamide and glycidamide; ETHOX, ethylene oxide; FORMAL, formaldehyde; UIO, iodine; PERNT, perchlorate, nitrate, and thiocyanate; UM, metals; PAH, polycyclic aromatic hydrocarbons; UHG, mercury; UVOC, volatile organic compounds; UTAS, total arsenic; UAS, speciated arsenics; UCOT, cotinine. ACR, acrylamide; GLY, glycidamide; EO, ethylene oxide; HCHO, formaldehyde; I, iodine; ClO_4^-, perchlorate; NO_3^-, nitrate; SCN^-, thiocyanate; Ba, barium; Cd, cadmium; Co, cobalt; Cs, cesium; Mo, molybdenum; Mn, manganese; Pb, lead; Sb, antimony; Sn, tin; Sr, strontium; Tl, thallium; W, tungsten; U, uranium; 1-OHN, 1-hydroxynaphthalene; 2-OHN, 2-hydroxynaphthalene; 3-OHFlu, 3-hydroxyfluorene; 2-OHFlu, 2-hydroxyfluorene; 1-OHPhe, 1-hydroxyphenanthrene; 2,3-OHPhe, 2- and 3-hydroxyphenanthrene; 1-OHP, 1-hydroxypyrene; Hg, mercury; 2-MHA, 2-methylhippuric acid; 3-MHA/4-MHA, 3-methylhippuric acid and 4-methylhippuric acid; NAc2CE-Cys, N-acetyl-S-(2-carbamoylethyl)-L-cysteine; NAcMC-Cys, N-acetyl-S-(N-methylcarbamoyl)-L-cysteine; 2-ATCA, 2-aminothiazoline-4-carboxylic acid; BMA, N-acetyl-S-(benzyl)-L-cysteine; NAcSP-Cys, N-acetyl-S-(n-propyl)-L-cysteine; CEMA, N-acetyl-S-(2-carboxyethyl)-L-cysteine; CYMA, N-acetyl-S-(2-cyanoethyl)-L-cysteine; DHBMA, N-acetyl-S-(3,4-dihydroxybutyl)-L-cysteine; NAc-2CHE-Cys, N-acetyl-S-(2-carbamoyl-2-hydroxyethyl)-L-cysteine; HEMA, N-acetyl-S-(2-hydroxyethyl)-L-cysteine; HPMA, N-acetyl-S-(2-hydroxypropyl)-L-cysteine; 3-HPMA, N-acetyl-S-(3-hydroxypropyl)-L-cysteine; MA, mandelic acid; MHBMA, N-acetyl-S-(4-hydroxy-2-butenyl)-L-cysteine; PhEMA, N-acetyl-S-(phenyl-2-hydroxyethyl)-L-cysteine; PGA, phenylglyoxylic acid; PHEMA, N-acetyl-S-(phenyl)-L-cysteine; NAc-3HPM-Cys, N-acetyl-S-(3-hydroxypropyl-1-methyl)-L-cysteine; As, total arsenic; As(III), arsenous acid; AsB, arsenobetaine; AsC, arsenocholine; DMA, dimethylarsinic acid; MMA, monomethylarsonic acid; COT, total cotinine; HCOT, total hydroxycotinine; TNE-2, total nicotine equivalent-2.

a Adjusted to control the false discovery rate at 5%.

Table S7. Mediation of Association Between Environmental Toxicants and HAZ by sex steroid hormones (TT, E2 and SHBG)

Family	Abbreviation	Mediator	ACME (95%CI)	P value (ACME) [a]	ADE (95%CI)	P value (ADE) [a]	P value (TE) [a]	Proportion of mediation
UVOC	2-ATCA	TT	-0.08 (-0.11, -0.06)	<0.001	-0.14 (-0.23, -0.05)	0.02	<0.001	0.37
UM	Cs	SHBG	-0.06 (-0.09, -0.04)	<0.001	-0.13 (-0.21, -0.04)	0.04	<0.001	0.33
UM	Mo	SHBG	-0.04 (-0.07, -0.03)	<0.001	-0.12 (-0.19, -0.05)	0.01	<0.001	0.27
UVOC	2-ATCA	SHBG	-0.06 (-0.08, -0.03)	<0.001	-0.16 (-0.25, -0.08)	<0.001	<0.001	0.25
UM	Mo	TT	-0.04 (-0.05, -0.02)	<0.001	-0.13 (-0.20, -0.05)	0.01	<0.001	0.22
UM	Pb	SHBG	-0.05 (-0.07, -0.03)	<0.001	-0.18 (-0.25, -0.11)	<0.001	<0.001	0.22
PERNT	NO3-	SHBG	-0.03 (-0.05, -0.01)	0	-0.12 (-0.21, -0.04)	0.02	<0.001	0.21
AMDGYD	GLY	TT	-0.02 (-0.04, -0.01)	<0.001	-0.09 (-0.16, -0.02)	0.05	<0.001	0.2
PAH	1-OHN	TT	-0.02 (-0.03, -0.01)	0	-0.07 (-0.13, -0.02)	0.05	0.01	0.2
UM	Cs	E2	-0.03 (-0.05, -0.02)	<0.001	-0.16 (-0.23, -0.07)	<0.001	<0.001	0.18
UM	Sn	SHBG	-0.04 (-0.07, -0.02)	<0.001	-0.22 (-0.31, -0.12)	<0.001	<0.001	0.17
UVOC	2-ATCA	E2	-0.03 (-0.05, -0.02)	<0.001	-0.19 (-0.27, -0.10)	<0.001	<0.001	0.16
UVOC	NAc-3HPM-Cys	E2	-0.01 (-0.03, -0.00)	0.01	-0.08 (-0.14, -0.02)	0.04	0.01	0.16
PERNT	NO3-	TT	-0.02 (-0.04, -0.01)	0	-0.13 (-0.21, -0.05)	0.01	<0.001	0.15
UM	Pb	TT	-0.04 (-0.05, -0.02)	<0.001	-0.20 (-0.26, -0.12)	<0.001	<0.001	0.15
UM	Sn	TT	-0.03 (-0.06, -0.01)	<0.001	-0.23 (-0.32, -0.14)	<0.001	<0.001	0.13
UM	Mo	E2	-0.02 (-0.04, -0.01)	<0.001	-0.14 (-0.21, -0.06)	<0.001	<0.001	0.13
PERNT	NO3-	E2	-0.02 (-0.03, -0.01)	0.01	-0.14 (-0.22, -0.06)	0.01	<0.001	0.12
UM	Pb	E2	-0.03 (-0.04, -0.01)	<0.001	-0.21 (-0.28, -0.13)	<0.001	<0.001	0.11
UM	Sn	E2	-0.03 (-0.04, -0.01)	<0.001	-0.23 (-0.32, -0.14)	<0.001	<0.001	0.1
UM	Sb	E2	-0.01 (-0.03, -0.00)	0.04	-0.14 (-0.20, -0.08)	<0.001	<0.001	0.09
UAS	MMA	TT	0.03 (0.00, 0.05)	0.04	-0.02 (-0.13, 0.09)	0.83	0.89	4.26

UAS	MMA	E2	0.02 (0.00, 0.04)	0.03	-0.02 (-0.12, 0.09)	0.85	0.9	3.53
PERNT	SCN-	E2	-0.02 (-0.03, -0.01)	0.01	0.01 (-0.07, 0.07)	0.9	0.75	1.42
UM	Co	SHBG	-0.06 (-0.09, -0.04)	<0.001	0.02 (-0.06, 0.09)	0.8	0.46	1.41
UVOC	NAc-2CHE-Cys	SHBG	0.07 (0.04, 0.11)	<0.001	-0.02 (-0.12, 0.09)	0.9	0.45	1.27
PAH	1-OHPhe	TT	-0.02 (-0.04, -0.01)	<0.001	0.00 (-0.06, 0.06)	0.92	0.65	1.25
UVOC	PHEMA	SHBG	0.04 (0.01, 0.07)	0.01	-0.01 (-0.11, 0.09)	0.96	0.65	1.21
PAH	2-OHN	SHBG	0.03 (0.01, 0.05)	<0.001	-0.00 (-0.08, 0.07)	0.96	0.59	1.18
UVOC	PGA	TT	-0.02 (-0.03, -0.01)	0.01	0.00 (-0.06, 0.06)	0.99	0.65	1.04
PAH	2-OHFlu	TT	-0.02 (-0.03, -0.00)	0.03	-0.00 (-0.06, 0.06)	0.99	0.68	0.93
UVOC	3-MHA/4-MHA	TT	-0.02 (-0.04, -0.01)	0.01	-0.00 (-0.07, 0.06)	0.99	0.64	0.85
PAH	1-OHPhe	E2	-0.02 (-0.03, -0.00)	0.01	-0.00 (-0.06, 0.06)	0.89	0.63	0.81
PAH	2-OHFlu	E2	-0.01 (-0.02, -0.00)	0.02	-0.01 (-0.06, 0.05)	0.89	0.63	0.71
UM	Mn	SHBG	0.03 (0.01, 0.07)	0.04	0.02 (-0.09, 0.13)	0.83	0.51	0.62
PAH	2,3-OHPhe	TT	-0.02 (-0.03, -0.00)	0.03	-0.01 (-0.07, 0.05)	0.81	0.5	0.59
UIO	I	SHBG	-0.04 (-0.06, -0.02)	<0.001	-0.03 (-0.11, 0.05)	0.62	0.18	0.56
UVOC	2-MHA	TT	-0.02 (-0.04, -0.01)	0.02	-0.02 (-0.08, 0.04)	0.74	0.44	0.54
UVOC	DHBMA	TT	-0.03 (-0.05, -0.01)	<0.001	-0.03 (-0.09, 0.04)	0.57	0.21	0.49
UCOT	COT	E2	-0.01 (-0.03, -0.00)	0.05	-0.02 (-0.09, 0.06)	0.74	0.51	0.47
UM	Tl	SHBG	-0.03 (-0.05, -0.01)	0.02	-0.04 (-0.12, 0.04)	0.6	0.21	0.47
PERNT	ClO4-	TT	-0.04 (-0.06, -0.02)	<0.001	-0.05 (-0.12, 0.03)	0.43	0.07	0.45
UVOC	MA	TT	-0.03 (-0.04, -0.01)	<0.001	-0.04 (-0.09, 0.02)	0.45	0.1	0.43
UVOC	DHBMA	SHBG	-0.02 (-0.05, -0.01)	0.01	-0.03 (-0.10, 0.03)	0.52	0.18	0.43
UVOC	NAcMC-Cys	TT	-0.02 (-0.03, -0.00)	0.03	-0.02 (-0.08, 0.03)	0.57	0.28	0.41
UVOC	3-HPMA	TT	-0.02 (-0.03, -0.01)	0.01	-0.03 (-0.09, 0.03)	0.57	0.28	0.41
UIO	I	TT	-0.03 (-0.05, -0.01)	<0.001	-0.04 (-0.13, 0.03)	0.45	0.17	0.38
UCOT	TNE-2	E2	-0.00 (-0.00, -0.00)	0.04	-0.00 (-0.00, 0.00)	0.66	0.46	0.38

PERNT	ClO4-	SHBG	-0.03 (-0.05, -0.01)	<0.001	-0.05 (-0.13, 0.02)	0.31	0.06	0.38
UM	Cs	TT	-0.07 (-0.09, -0.04)	<0.001	-0.12 (-0.21, -0.03)	0.05	0.01	0.36
UAS	AsB	SHBG	0.05 (0.02, 0.08)	<0.001	0.10 (-0.02, 0.21)	0.3	0.05	0.36
UVOC	MHBMA	TT	-0.03 (-0.05, -0.02)	<0.001	-0.06 (-0.12, 0.00)	0.19	0.02	0.34
UVOC	NAc-3HPM-Cys	TT	-0.03 (-0.05, -0.02)	<0.001	-0.06 (-0.13, -0.00)	0.13	0.01	0.33
UCOT	HCOT	E2	-0.01 (-0.03, -0.00)	0.04	-0.03 (-0.11, 0.04)	0.63	0.43	0.33
UVOC	MA	SHBG	-0.02 (-0.04, -0.01)	0.02	-0.04 (-0.10, 0.02)	0.36	0.09	0.33
UM	Tl	TT	-0.02 (-0.04, -0.00)	0.04	-0.05 (-0.13, 0.03)	0.46	0.22	0.32
UM	W	E2	-0.02 (-0.03, -0.01)	0.01	-0.04 (-0.11, 0.03)	0.5	0.21	0.32
UVOC	DHBMA	E2	-0.02 (-0.03, -0.01)	0	-0.04 (-0.11, 0.03)	0.5	0.21	0.32
UVOC	NAc-3HPM-Cys	SHBG	-0.03 (-0.05, -0.01)	<0.001	-0.06 (-0.12, -0.01)	0.13	0.01	0.32
UVOC	3-HPMA	E2	-0.01 (-0.03, -0.00)	0.01	-0.03 (-0.10, 0.03)	0.5	0.26	0.31
UVOC	CYMA	E2	-0.02 (-0.02, -0.01)	<0.001	-0.04 (-0.08, 0.01)	0.31	0.07	0.3
PERNT	ClO4-	E2	-0.02 (-0.04, -0.01)	<0.001	-0.06 (-0.14, 0.01)	0.22	0.06	0.28
UAS	DMA	SHBG	-0.02 (-0.04, -0.01)	0.02	-0.06 (-0.13, 0.01)	0.26	0.05	0.28
AMDGYD	ACR	E2	-0.02 (-0.03, -0.01)	0	-0.05 (-0.10, 0.01)	0.25	0.08	0.25
AMDGYD	GLY	E2	-0.03 (-0.04, -0.02)	<0.001	-0.08 (-0.15, -0.01)	0.06	0.01	0.25
PAH	3-OHFlu	SHBG	-0.02 (-0.03, -0.00)	0.03	-0.05 (-0.11, 0.01)	0.23	0.05	0.25
UVOC	MHBMA	SHBG	-0.02 (-0.04, -0.01)	0.02	-0.06 (-0.12, -0.00)	0.11	0.01	0.25
UM	Cd	TT	0.04 (0.01, 0.06)	<0.001	0.12 (0.01, 0.22)	0.13	0.04	0.24
UVOC	HPMA	TT	-0.02 (-0.03, -0.01)	<0.001	-0.07 (-0.13, -0.01)	0.08	0.01	0.23
UAS	DMA	TT	-0.02 (-0.04, -0.00)	0.03	-0.07 (-0.14, 0.00)	0.15	0.05	0.23
UM	Cd	SHBG	0.03 (0.01, 0.06)	0.02	0.12 (0.01, 0.22)	0.1	0.02	0.23
UVOC	MHBMA	E2	-0.02 (-0.03, -0.01)	<0.001	-0.07 (-0.13, -0.01)	0.09	0.02	0.22
PAH	3-OHFlu	E2	-0.01 (-0.03, -0.00)	0.01	-0.06 (-0.12, 0.00)	0.18	0.05	0.2
UVOC	HPMA	E2	-0.02 (-0.03, -0.01)	<0.001	-0.07 (-0.13, -0.02)	0.06	0.01	0.2

UVOC	MA	E2	-0.01 (-0.02, -0.00)	0.02	-0.05 (-0.11, 0.01)	0.27	0.1	0.2
ETHOX	EO	E2	-0.01 (-0.02, -0.00)	0.01	-0.06 (-0.12, -0.01)	0.06	0.02	0.18
UM	Cd	E2	0.02 (0.01, 0.04)	0.02	0.13 (0.02, 0.24)	0.07	0.02	0.16
UAS	DMA	E2	-0.01 (-0.03, -0.00)	0.05	-0.07 (-0.15, 0.00)	0.16	0.07	0.15
UM	Co	E2	0.02 (0.01, 0.03)	0.01	-0.06 (-0.14, 0.02)	0.31	0.47	-0.39
UM	U	TT	0.02 (0.00, 0.03)	0.02	-0.05 (-0.12, 0.02)	0.36	0.5	-0.53
UVOC	NAcSP-Cys	SHBG	0.04 (0.01, 0.07)	0.01	-0.06 (-0.17, 0.05)	0.47	0.78	-2.41

Abbreviations: CI, confidence interval; ACME, average causal mediation effect; ADE, average direct effect; TE, total effect;AMDGYD, acrylamide and glycidamide; ETHOX, ethylene oxide; FORMAL, formaldehyde; UIO, iodine; PERNT, perchlorate, nitrate, and thiocyanate; UM, metals; PAH, polycyclic aromatic hydrocarbons; UHG, mercury; UVOC, volatile organic compounds; UTAS, total arsenic; UAS, speciated arsenics; UCOT, cotinine. ACR, acrylamide; GLY, glycidamide; EO, ethylene oxide; HCHO, formaldehyde; I, iodine; ClO_4^-, perchlorate; NO_3^-, nitrate; SCN^-, thiocyanate; Ba, barium; Cd, cadmium; Co, cobalt; Cs, cesium; Mo, molybdenum; Mn, manganese; Pb, lead; Sb, antimony; Sn, tin; Sr, strontium; Tl, thallium; W, tungsten; U, uranium; 1-OHN, 1-hydroxynaphthalene; 2-OHN, 2-hydroxynaphthalene; 3-OHFlu, 3-hydroxyfluorene; 2-OHFlu, 2-hydroxyfluorene; 1-OHPhe, 1-hydroxyphenanthrene; 2,3-OHPhe, 2- and 3-hydroxyphenanthrene; 1-OHP, 1-hydroxypyrene; Hg, mercury; 2-MHA, 2-methylhippuric acid; 3-MHA/4-MHA, 3-methylhippuric acid and 4-methylhippuric acid; NAc2CE-Cys, N-acetyl-S-(2-carbamoylethyl)-L-cysteine; NAcMC-Cys, N-acetyl-S-(N-methylcarbamoyl)-L-cysteine; 2-ATCA, 2-aminothiazoline-4-carboxylic acid; BMA, N-acetyl-S-(benzyl)-L-cysteine; NAcSP-Cys, N-acetyl-S-(n-propyl)-L-cysteine; CEMA, N-acetyl-S-(2-carboxyethyl)-L-cysteine; CYMA, N-acetyl-S-(2-cyanoethyl)-L-cysteine; DHBMA, N-acetyl-S-(3,4-dihydroxybutyl)-L-cysteine; NAc-2CHE-Cys, N-acetyl-S-(2-carbamoyl-2-hydroxyethyl)-L-cysteine; HEMA, N-acetyl-S-(2-hydroxyethyl)-L-cysteine; HPMA, N-acetyl-S-(2-hydroxypropyl)-L-cysteine; 3-HPMA, N-acetyl-S-(3-hydroxypropyl)-L-cysteine; MA, mandelic acid; MHBMA, N-acetyl-S-(4-hydroxy-2-butenyl)-L-cysteine; PhEMA, N-acetyl-S-(phenyl-2-hydroxyethyl)-L-cysteine; PGA, phenylglyoxylic acid; PHEMA, N-acetyl-S-(phenyl)-L-cysteine; NAc-3HPM-Cys, N-acetyl-S-(3-hydroxypropyl-1-methyl)-L-cysteine; As, total arsenic; As(III), arsenous acid; AsB, arsenobetaine; AsC, arsenocholine; DMA, dimethylarsinic acid; MMA, monomethylarsonic acid; COT, total cotinine; HCOT, total hydroxycotinine; TNE-2, total nicotine equivalent-2.

a Adjusted to control the false discovery rate at 5%.

Figure S1. Flowchart of Sample Selection

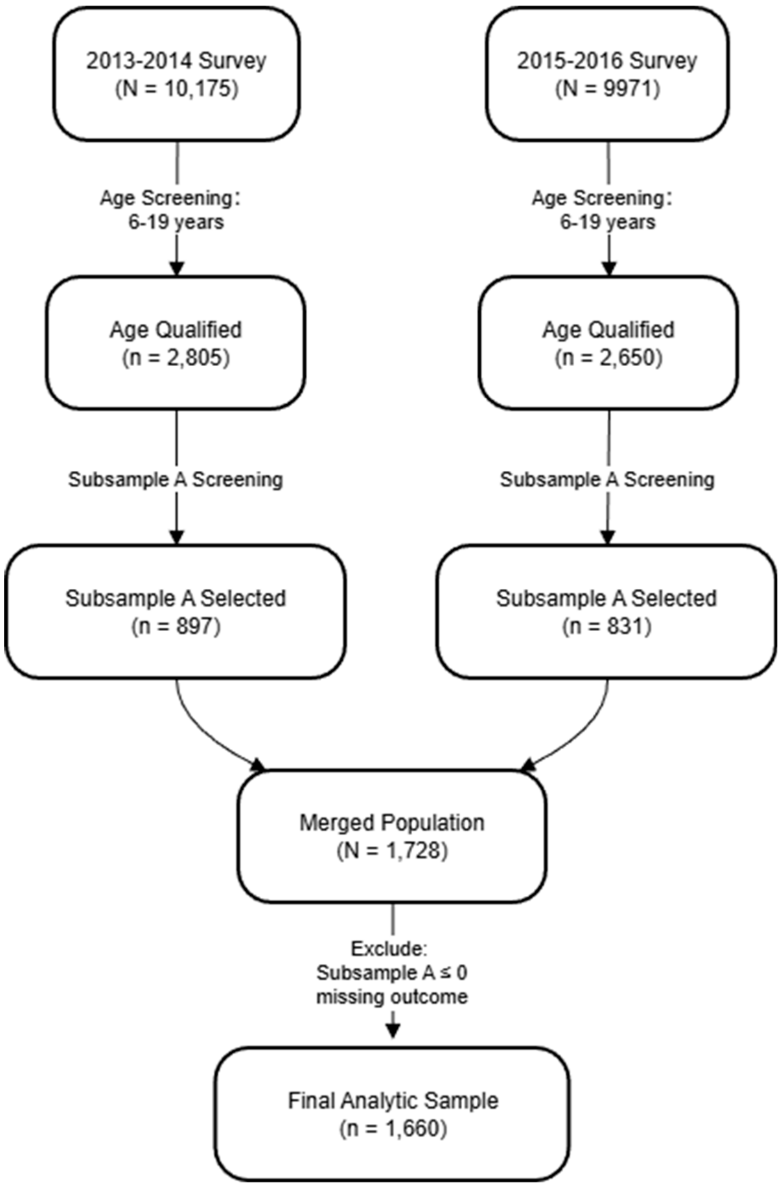

Figure S2. Correlation of the Environmental Toxicants (58 Exposures)

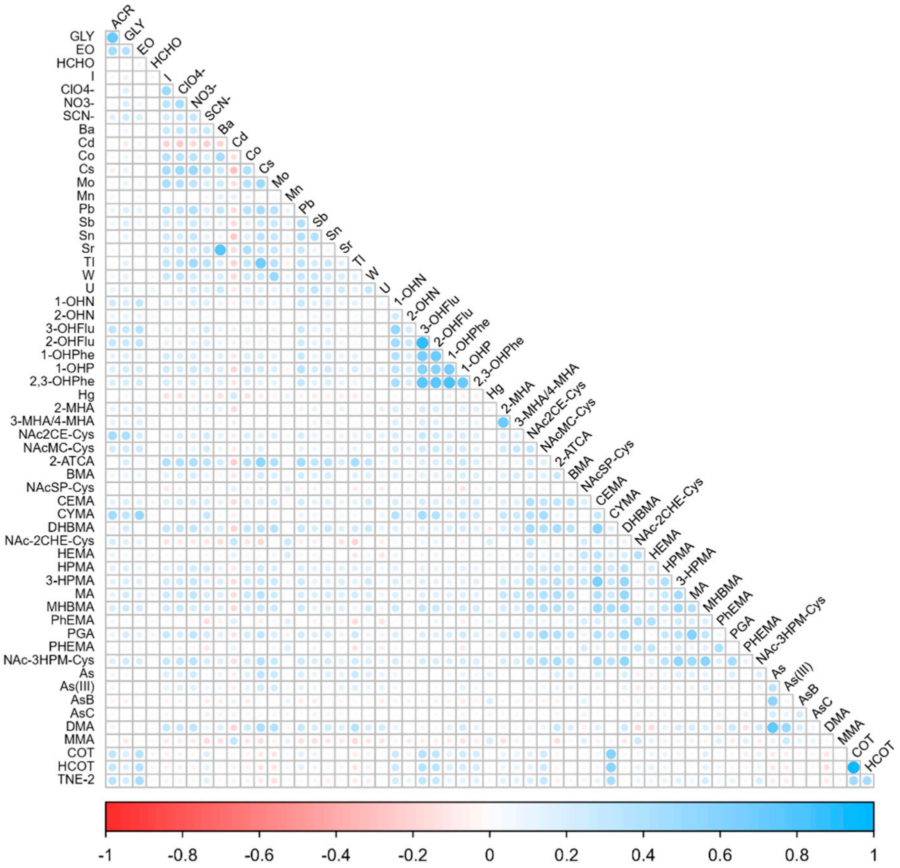

Abbreviations: ACR, acrylamide; GLY, glycidamide; EO, ethylene oxide; HCHO, formaldehyde; I, iodine; ClO_4^-, perchlorate; NO_3^-, nitrate; SCN^-, thiocyanate; Ba, barium; Cd, cadmium; Co, cobalt; Cs, cesium; Mo, molybdenum; Mn, manganese; Pb, lead; Sb, antimony; Sn, tin; Sr, strontium; Tl, thallium; W, tungsten; U, uranium; 1-OHN, 1-hydroxynaphthalene; 2-OHN, 2-hydroxynaphthalene; 3-OHFlu, 3-hydroxyfluorene; 2-OHFlu, 2-hydroxyfluorene; 1-OHPhe, 1-hydroxyphenanthrene; 2,3-OHPhe, 2- and 3-hydroxyphenanthrene; 1-OHP, 1-hydroxypyrene; Hg, mercury; 2-MHA, 2-methylhippuric acid; 3-MHA/4-MHA, 3-methylhippuric acid and 4-methylhippuric acid; NAc2CE-Cys, N-acetyl-S-(2-carbamoylethyl)-L-cysteine; NAcMC-Cys, N-acetyl-S-(N-methylcarbamoyl)-L-cysteine; 2-ATCA, 2-aminothiazoline-4-carboxylic acid; BMA, N-acetyl-S-(benzyl)-L-cysteine; NAcSP-Cys, N-acetyl-S-(n-propyl)-L-cysteine; CEMA, N-acetyl-S-(2-carboxyethyl)-L-cysteine; CYMA, N-acetyl-S-(2-cyanoethyl)-L-cysteine; DHBMA, N-acetyl-S-(3,4-dihydroxybutyl)-L-cysteine; NAc-2CHE-Cys, N-acetyl-S-(2-carbamoyl-2-hydroxyethyl)-L-cysteine; HEMA, N-acetyl-S-(2-hydroxyethyl)-L-cysteine; HPMA, N-acetyl-S-(2-hydroxypropyl)-L-cysteine; 3-HPMA, N-acetyl-S-(3-hydroxypropyl)-L-cysteine; MA, mandelic acid; MHBMA, N-acetyl-S-(4-hydroxy-2-butenyl)-L-cysteine; PhEMA, N-acetyl-S-(phenyl-2-hydroxyethyl)-L-cysteine; PGA, phenylglyoxylic acid; PHEMA, N-acetyl-S-(phenyl)-L-cysteine; NAc-3HPM-Cys, N-acetyl-S-(3-hydroxypropyl-1-methyl)-L-cysteine; As, total arsenic; As(III), arsenous acid; AsB, arsenobetaine; AsC, arsenocholine; DMA, dimethylarsinic acid; MMA, monomethylarsonic acid; COT, total cotinine; HCOT, total hydroxycotinine; TNE-2, total nicotine equivalent-2.

Figure S3. Sensitivity Analysis of Associations Between an Expanded Set of 65 Environmental Toxicants and Height-for-Age Z-Score (HAZ).

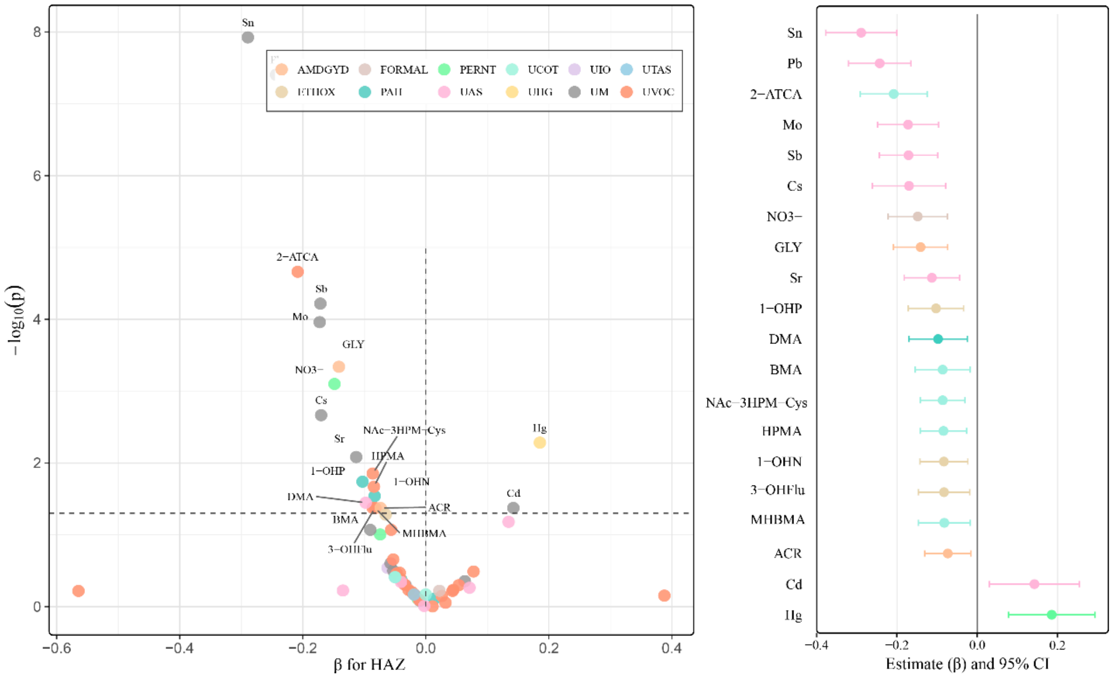

Notes: The left panel displays a volcano plot illustrating the associations between toxicants and HAZ. The horizontal dashed line represents the statistical significance threshold after adjusting for the false discovery rate (FDR < 0.05), and the vertical dashed line indicates an estimate (β) of zero. The right panel shows the estimated β coefficients and 95%Cis for toxicants with significant associations (P value < 0.05). All results were derived from Model 2, adjusted for age, sex, race/ethnicity, daily energy intake, weekly physical activity frequency, household smokers, and family income-to-poverty ratio.

Abbreviations: AMDGYD, acrylamide and glycidamide; ETHOX, ethylene oxide; FORMAL, formaldehyde; PERNT, perchlorate, nitrate, and thiocyanate; UCOT, cotinine; UIO, iodine; UTAS, total arsenic; UAS, speciated arsenics; UHG, mercury; UM, metals; PAH, polycyclic aromatic hydrocarbons; UVOC, volatile organic compounds; Sn, tin; Pb, lead; 2-ATCA, 2-aminothiazoline-4-carboxylic acid; Cs, cesium; Mo, molybdenum; Sb, antimony; NO_3^-, nitrate; GLY, glycidamide; Sr, strontium; 1-OHP, 1-hydroxypyrene; NAc-3HPM-Cys, N-acetyl-S-(3-hydroxypropyl-1-methyl)-L-cysteine; BMA, N-acetyl-S-(benzyl)-L-cysteine; HPMA, N-acetyl-S-(2-hydroxypropyl)-L-cysteine; 1-OHN, 1-hydroxynaphthalene; MHBMA, N-acetyl-S-(4-hydroxy-2-butenyl)-L-cysteine; ACR, acrylamide; Cd, cadmium; Hg, mercury .

Figure S4 Linear Dose-Response Relationship Between the Cumulative Weighted Quantile Sum (WQS) Index and Covariate-Adjusted HAZ.

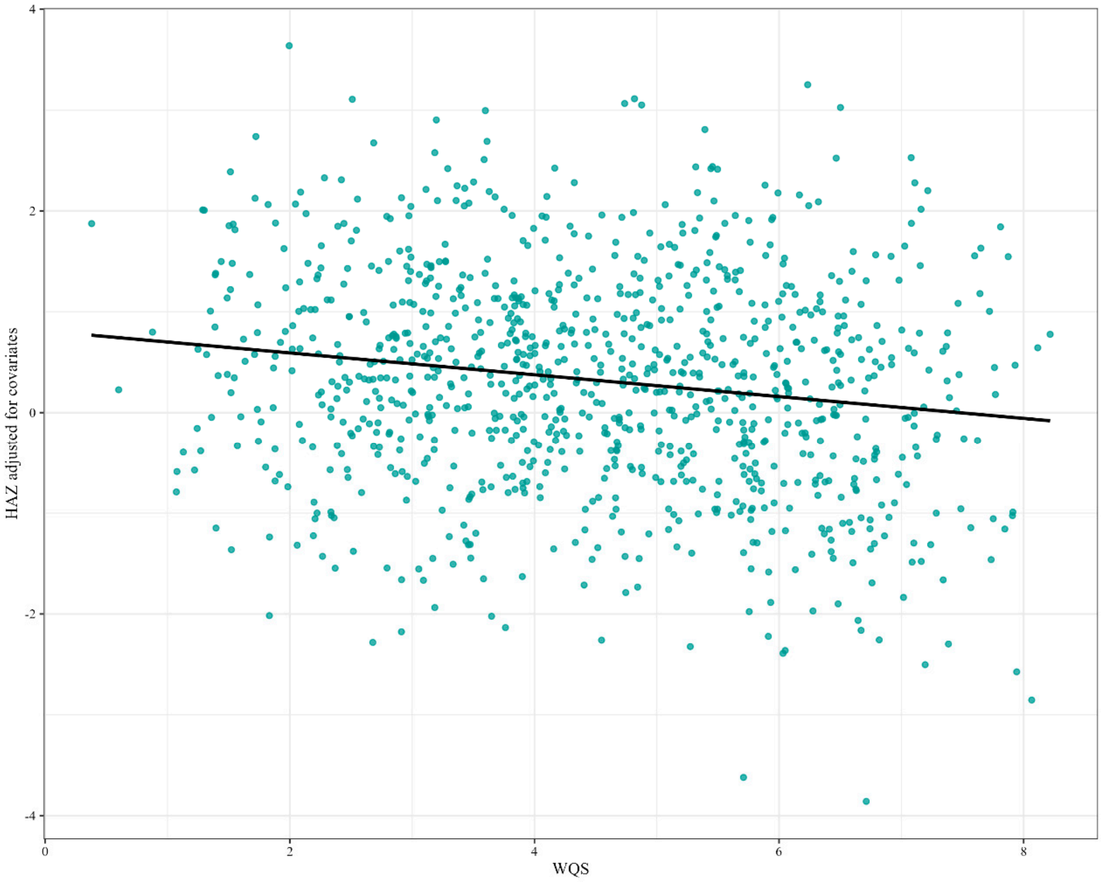

Notes: The scatter plot visualizes the cumulative effect of the multipollutant mixture on linear growth. The x-axis represents the WQS index, constructed from the bootstrapped weights of the continuous toxicant exposures. The y-axis represents the residual HAZ values after partialling out the effects of covariates. The solid black line indicates the fitted linear regression trend, demonstrating an inverse association between the overall toxicant mixture burden and height development. The model was constrained to a negative direction to evaluate joint adverse effects and was fully adjusted for age, sex, race/ethnicity, daily energy intake, weekly physical activity frequency, number of household smokers, and family income-to-poverty ratio.

Abbreviations: HAZ, Height-for-Age Z-Score; WQS, Weighted Quantile Sum.

Figure S5 Nonlinear exposure–response curve in BKMR model

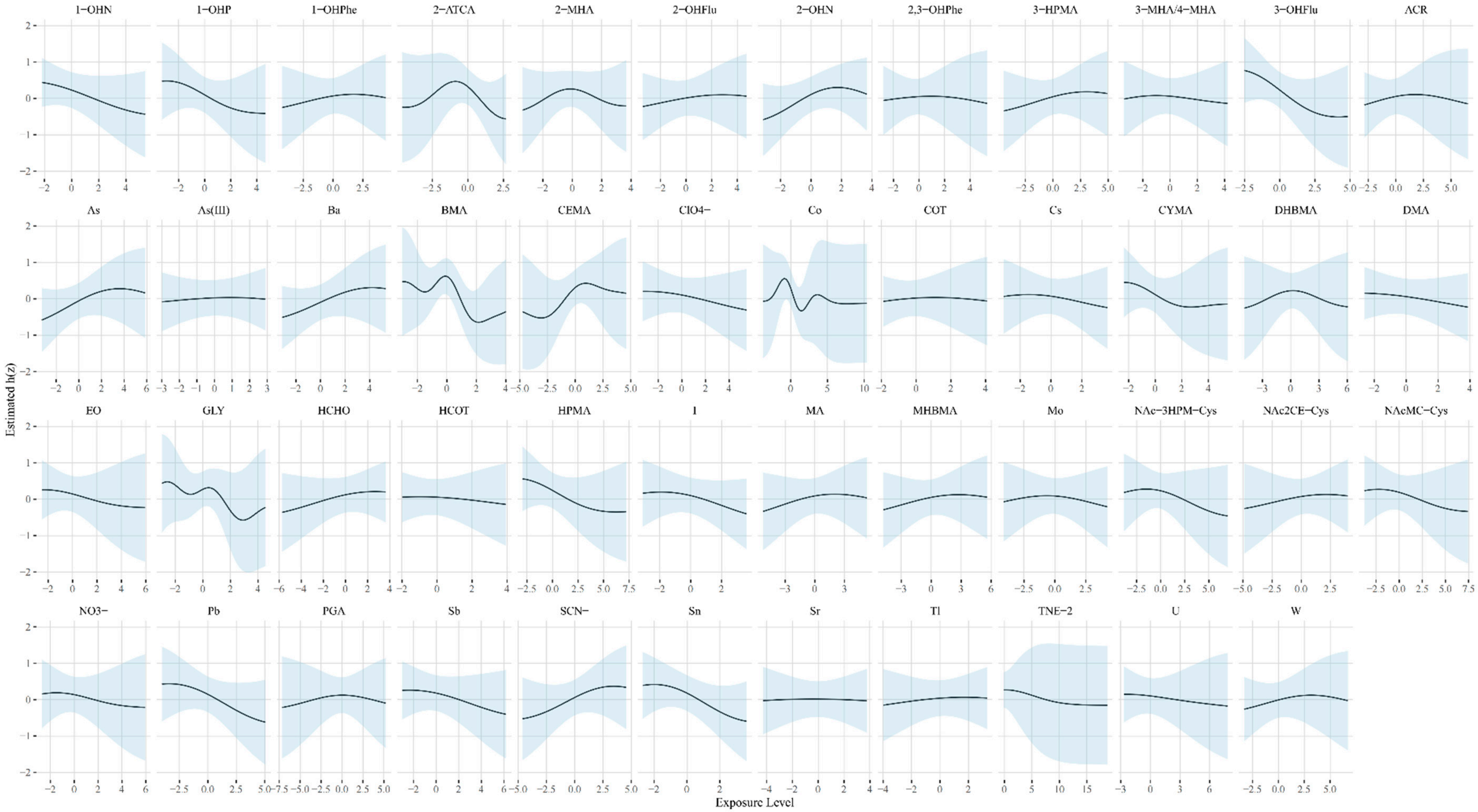

Abbreviations: ACR, acrylamide; GLY, glycidamide; EO, ethylene oxide; HCHO, formaldehyde; I, iodine; ClO_4^-, perchlorate; NO_3^-, nitrate; SCN^-, thiocyanate; Ba, barium; Cd, cadmium; Co, cobalt; Cs, cesium; Mo, molybdenum; Mn, manganese; Pb, lead; Sb, antimony; Sn, tin; Sr, strontium; Tl, thallium; W, tungsten; U, uranium; 1-OHN, 1-hydroxynaphthalene; 2-OHN, 2-hydroxynaphthalene; 3-OHFlu, 3-hydroxyfluorene; 2-OHFlu, 2-hydroxyfluorene; 1-OHPhe, 1-hydroxyphenanthrene; 2,3-OHPhe, 2- and 3-hydroxyphenanthrene; 1-OHP, 1-hydroxypyrene; Hg, mercury; 2-MHA, 2-methylhippuric acid; 3-MHA/4-MHA, 3-methylhippuric acid and 4-methylhippuric acid; NAc2CE-Cys, N-acetyl-S-(2-carbamoylethyl)-L-cysteine; NAcMC-Cys, N-acetyl-S-(N-methylcarbamoyl)-L-cysteine; 2-ATCA, 2-aminothiazoline-4-carboxylic acid; BMA, N-acetyl-S-(benzyl)-L-cysteine; NAcSP-Cys, N-acetyl-S-(n-propyl)-L-cysteine; CEMA, N-acetyl-S-(2-carboxyethyl)-L-cysteine; CYMA, N-acetyl-S-(2-cyanoethyl)-L-cysteine; DHBMA, N-acetyl-S-(3,4-dihydroxybutyl)-L-cysteine; NAc-2CHE-Cys, N-acetyl-S-(2-carbamoyl-2-hydroxyethyl)-L-cysteine; HEMA, N-acetyl-S-(2-hydroxyethyl)-L-cysteine; HPMA, N-acetyl-S-(2-hydroxypropyl)-L-cysteine; 3-HPMA, N-acetyl-S-(3-hydroxypropyl)-L-cysteine; MA, mandelic acid; MHBMA, N-acetyl-S-(4-hydroxy-2-butenyl)-L-cysteine; PhEMA, N-acetyl-S-(phenyl-2-hydroxyethyl)-L-cysteine; PGA, phenylglyoxylic acid; PHEMA, N-acetyl-S-(phenyl)-L-cysteine; NAc-3HPM-Cys, N-acetyl-S-(3-hydroxypropyl-1-methyl)-L-cysteine; As, total arsenic; As(III), arsenous acid; AsB, arsenobetaine; AsC, arsenocholine; DMA, dimethylarsinic acid; MMA, monomethylarsonic acid; COT, total cotinine; HCOT, total hydroxycotinine; TNE-2, total nicotine equivalent-2.

References

(1) Johnson, C. L.; Dohrmann, S. M.; Burt, V. L.; Mohadjer, L. K. National health and nutrition examination survey: sample design, 2011-2014. *Vital Health Stat 2* 2014, (162), 1-33.

(2) Centers for Disease Control and Prevention (CDC). *Laboratory Data Overview of NHANES 2013-2014* https://wwwn.cdc.gov/nchs/nhanes/continuousnhanes/overviewlab.aspx?BeginYear=2013 (accessed May 28, 2025).

(3) Centers for Disease Control and Prevention (CDC). *Laboratory Data Overview of NHANES 2015-2016.* https://wwwn.cdc.gov/nchs/nhanes/continuousnhanes/overviewlab.aspx?BeginYear=2015. (accessed May 28, 2025).

(4) Benjamini, Y.; Hochberg, Y. Controlling the False Discovery Rate: A Practical and Powerful Approach to Multiple Testing. *Journal of the Royal Statistical Society: Series B (Methodological)* 2018, *57* (1), 289-300. DOI: 10.1111/j.2517-6161.1995.tb02031.x (acccessed 12/22/2025).

(5) Carrico, C.; Gennings, C.; Wheeler, D. C.; Factor-Litvak, P. Characterization of Weighted Quantile Sum Regression for Highly Correlated Data in a Risk Analysis Setting. *J Agric Biol Environ Stat* 2015, *20* (1), 100-120. DOI: 10.1007/s13253-014-0180-3

(6) Bobb, J. F.; Valeri, L.; Claus Henn, B.; Christiani, D. C.; Wright, R. O.; Mazumdar, M.; Godleski, J. J.; Coull, B. A. Bayesian kernel machine regression for estimating the health effects of multi-pollutant mixtures. *Biostatistics* 2015, *16* (3), 493-508. DOI: 10.1093/biostatistics/kxu058

(7) Imai, K.; Keele, L.; Tingley, D. A general approach to causal mediation analysis. *Psychol Methods* 2010, *15* (4), 309-334. DOI: 10.1037/a0020761
